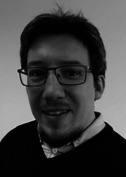# Sustainable Battery Materials from Biomass

**DOI:** 10.1002/cssc.201903577

**Published:** 2020-04-15

**Authors:** Clemens Liedel

**Affiliations:** ^1^ Department Colloid Chemistry Max Planck Institute of Colloids and Interfaces Am Mühlenberg 1 14476 Potsdam Germany

**Keywords:** biomass, charge storage, electrode materials, energy storage, organic batteries

## Abstract

Sustainable sources of energy have been identified as a possible way out of today's oil dependency and are being rapidly developed. In contrast, storage of energy to a large extent still relies on heavy metals in batteries. Especially when built from biomass‐derived organics, organic batteries are promising alternatives and pave the way towards truly sustainable energy storage. First described in 2008, research on biomass‐derived electrodes has been taken up by a multitude of researchers worldwide. Nowadays, in principle, electrodes in batteries could be composed of all kinds of carbonized and noncarbonized biomass: On one hand, all kinds of (waste) biomass may be carbonized and used in anodes of lithium‐ or sodium‐ion batteries, cathodes in metal–sulfur or metal–oxygen batteries, or as conductive additives. On the other hand, a plethora of biomolecules, such as quinones, flavins, or carboxylates, contain redox‐active groups that can be used as redox‐active components in electrodes with very little chemical modification. Biomass‐based binders can replace toxic halogenated commercial binders to enable a truly sustainable future of energy storage devices. Besides the electrodes, electrolytes and separators may also be synthesized from biomass. In this Review, recent research progress in this rapidly emerging field is summarized with a focus on potentially fully biowaste‐derived batteries.

## Graphical abstract

**So the storage goes**: Biomass‐derived energy storage devices are attracting increasing attention. Waste biomass may be carbonized and used in electrodes for lithium‐ion, sodium‐ion batteries, metal–sulfur, or metal–oxygen batteries, or as conductive additives. Moreover, many biomolecules containing redox‐active groups can be used in electrodes with very little chemical modification. In this Review, recent research progress in this rapidly emerging field is summarized.

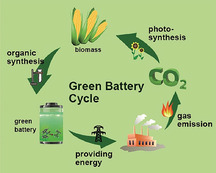

## 1 Introduction

In 2008, Chen et al. presented dilithium rhodizonate as a biomass‐derived sustainable cathode material for lithium‐ion batteries with a high charge storage capability at a reasonable potential. They foresaw that the “consideration of renewable resources in designing electrode materials could potentially enable the realization of green and sustainable batteries within the next decade.”^1^ Since then, significant advancements have been made, and several concepts of green and sustainable batteries have been presented. Now, more than one decade later, it is time to evaluate developments and future trends in the field of battery materials made from renewable resources. This Review will summarize major accomplishments and give an outlook to future sustainable biomass‐derived batteries.

The need for renewable sources of energy is well‐known and has long been identified as a possible way out of today's oil dependency.^2^ For truly sustainable usage of renewable energy, however, devices for energy storage should also be as benign as possible, for example, by being made of sustainable materials. In contrast to nonrenewable sources of energy (chemicals that release energy upon burning), which can rather easily be stored in tanks and used when needed, renewable sources of energy predominantly produce electrical energy, which requires more sophisticated storage devices. Importantly, not only off‐grid devices, such as cellphones and cars, necessitate such advanced devices for the storage of electrical energy, but the fluctuating availability of renewable electrical energy depending on the weather or time of day (in the case of windmills and solar panels, respectively) additionally demands for advanced grid storage of electrical energy. Common storage devices in this regard are supercapacitors and batteries, with batteries usually enabling higher energy density at the expense of power density.

When talking about sustainable battery materials, the concept of sustainability in chemistry needs to be discussed first.^3^ It includes not only the principles of green chemistry, as introduced by Anastas and Warner,^4^ but also aspects like water purification, alternative energies, exposure control of chemicals, and others.^5^ In general, chemistry can only be considered sustainable if—adapted from a United Nations definition of sustainable development—it “meets the needs of the present without compromising the ability of future generations to meet their own needs”;^6^ that is, fossil resources are not being depleted, the environment is not being polluted, and feedstocks are completely renewable and not overused. A sustainable chemical process should be environmentally benign, economical in its use of resources, techniques, and industrial feasibility, and socially responsible, whereas a sustainable chemical material should be environmentally benign throughout its full lifecycle, including mining, usage, and recycling. It should furthermore be economical during fabrication, distribution, usage, and recycling, as well as being produced and used in a socially responsible way. To some extent, side products of contemporary industries, such as sulfur as a waste product from petrochemical industry, might also qualify as rather sustainable raw materials.

Larcher and Tarascon discussed the concept of sustainability for batteries.^7^ The impact of a cell not only depends on the chemical composition but is the sum of the impacts of chemical composition, synthesis process, implementation in the system, and recycling. Commercial lithium‐ion batteries usually fail such sustainability criteria. They typically comprise powdered heavy metal‐containing inorganic active materials in the electrodes (often obtained under questionable conditions in developing countries), very thin membranes to separate cathode from anode, and highly flammable carbonate‐based electrolytes that form a resistive solid–electrolyte interphase (SEI) on the electrodes, leading to heat generation in operation.^8^ This setup inherently imposes dangers during malfunction, and even though the always‐implemented battery monitoring system usually prevents problems during operation, battery fires are omnipresent problems that can often be encountered in the media. Moreover, recycling of lithium‐ion batteries is not a widely established process yet,^9^ and production as well as operation results in significant greenhouse gas emissions.^10^

Many approaches have been described for increasing the sustainability of battery materials, which have usually tackled individual aspects such as composition,^8^, ^11^ recycling,^12^ and implementation. For example, intense efforts have been made to replace cobalt in LiCoO_2_ cathodes by more abundant elements because of socio‐economic and ecological concerns, as well as limited supply and limited full cell potential. Higher cell potential alongside more sustainable cathode materials have been achieved for example by moving from layered oxides, as in LiCoO_2_, towards certain phospho‐olivines or spinel oxides. These more available inorganic cathode (and also anode) materials may be obtained by using biotemplates,^13^, ^14^, ^15^ biomineralization,^16^ and other low‐temperature processes. These sustainable batteries with inorganic electrodes have been summarized in recent years in several excellent reviews.^11^, ^17^

Organic materials may similarly be used instead of common inorganic electrode materials and have been investigated for several decades now.^18^ Using any organics as active cell components decreases the need for rare metals and, as such, contributes to more sustainable energy storage. Thus, in many reports, organic electrode materials are *per se* denoted “green” or “sustainable”. A plethora of reviews have summarized recent trends in organic electrode materials.^19^, ^20^, ^21^, ^22^, ^23^, ^24^, ^25^, ^26^, ^27^, ^28^, ^29^, ^30^, ^31^, ^32^, ^33^, ^34^, ^35^, ^36^, ^37^, ^38^, ^39^, ^40^, ^41^, ^42^, ^43^ Most reports however do not focus on biomass‐based materials but describe materials derived from petrochemicals. Although fossil oil and gas have been formed from biomass a long time ago, their supply is limited on a shorter timescale. Hence, truly sustainable organic materials should not rely on petrochemicals but be made from regrown resources. Most reports about organic electrodes, however, which describe sophisticated new compounds, usually made from oil‐based chemistry, fail the aforementioned definition of sustainable chemistry. More sustainable organics should be desirable, but, in many reports, concerns about sustainability are subordinate to performance.

In general, organic electrode materials for batteries may be classified as n‐type (in which the neutral state can be reduced to a negatively charged state), p‐type (in which the neutral state can be oxidized to a positively charged state), or b‐type (in which the neutral state can both be reduced to the negatively charged state and oxidized to the positively charged state), depending on their redox reactions. In principle, n‐, p‐, and b‐type organics may be used as cathode or anode material. Because of stability reasons and redox potential, p‐type organics however are only used in organic cathodes, whereas n‐ and b‐type organics may be used in both electrodes.^21^ In nature, n‐type redox reactions are more common than p‐type redox reactions, meaning that biomolecules can, in principle, be used in both electrodes of a battery.

Another means by which to classify organic electrode materials is by their redox chemistry. Different redox mechanisms are present in conjugated systems, carbonyl compounds, stable radical containing compounds, organodisulfides, and thioethers. Out of these, only some carbonyl and possibly some sulfur‐containing compounds are directly available from biomass. All other classes of material need to be synthesized from petrochemical precursors, or require harsh, unsustainable modifications of biomass‐derived chemicals and are, as such, not as sustainable as chemicals that are directly available in regrown biomass or can be synthesized from biomass in benign reactions.

**Figure 1:**
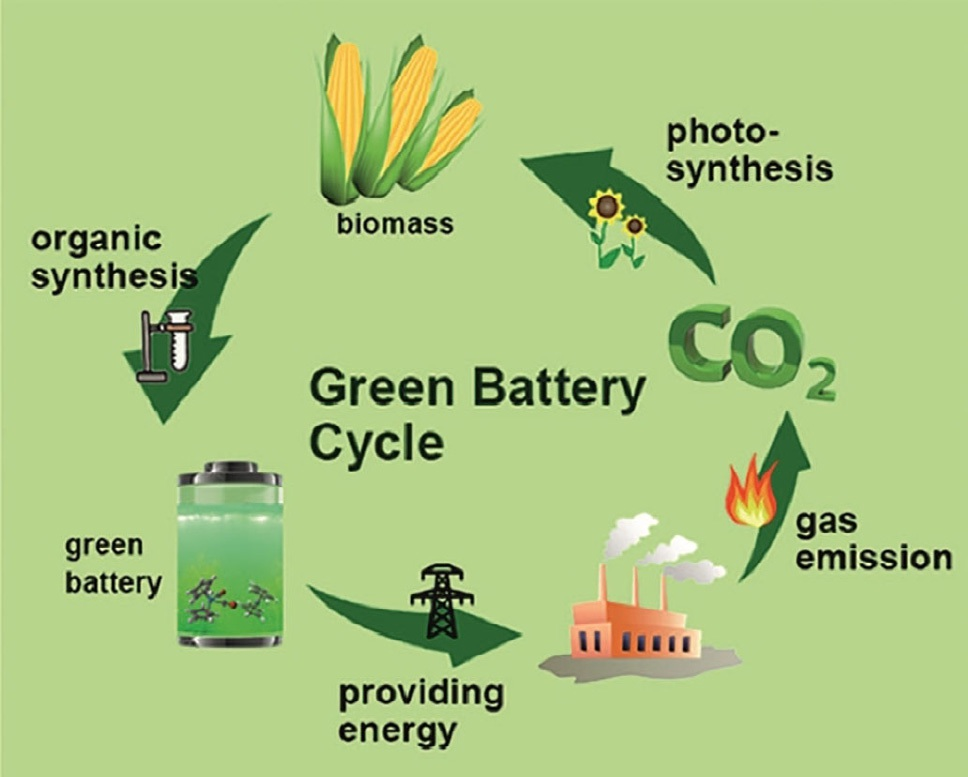
Ideal green battery cycle. Reproduced with permission from ref. 45. Copyright 2017 by Wiley‐VCH.

In this Review, organic battery components may only be considered sustainable if they can be made from biological resources in a sustainable way, and if they can be implemented in cells in a benign process. Chemicals made from oil or other fossil fuels shall not be considered bioderived herein, even though of course their feedstocks were also formed from biomass millions of years ago. Although nowadays a vast variety of small molecules can be produced from regrown biomass by using only biological reactions,^44^ only some are redox‐active and may hence find application in sustainable electrodes. Other biomass‐based small molecules may be used to synthesize different parts of sustainable batteries, such as binders or electrolytes. In energy storage devices relying on a combination of such materials, the full carbon cycle is maintained (Figure 1). Ideally, biomass‐based batteries power machines, which generate CO_2_, which is transformed into biomass in plants, which is used to make batteries again. Additionally, batteries that reached the end of their usability may be decomposed biologically or incinerated, releasing the constituents back to the environment to ideally form new biogenic chemicals in a natural way.

Research on truly sustainable organic electrode materials made a leap forward ten years ago, after Armand and Tarascon envisioned batteries made from renewable resources.^19^ Although the question whether a process can ever be completely sustainable in its strongest definition is of philosophical nature and cannot be answered here and complete sustainability of a battery with all its constituents and full lifecycle may never be reached, steps towards more sustainable organic batteries are necessary. Recent achievements regarding sustainable biomass‐based electrodes shall be reviewed in the following. Additionally, other biomass‐based constituents of sustainable batteries will be introduced.

## 2 Electrodes

### 2.1 Renewable carbon materials

**Figure 2:**
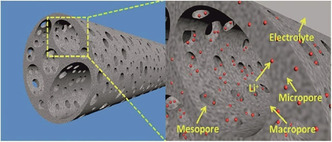
Schematic representation of hierarchical pores in hard carbons and transport behavior of electrolytes. Reproduced with permission from ref. 68. Copyright 2014 by the Royal Society of Chemistry.

Anodes in state‐of‐the‐art lithium‐ion batteries are based on lithium, which is intercalated in graphite during reduction of lithium ions (charging) and deintercalated upon oxidation (discharging). The main advantage of this arrangement is that it impedes the formation of dendrites. Furthermore, natural graphite is a still‐abundant resource, which, even in battery grade, is usually cheaper than carbon materials synthesized in a chemical laboratory, even though all kinds of (waste) biomass may be carbonized, as described in many reviews.^24^, ^46^, ^47^, ^48^, ^49^, ^50^, ^51^, ^52^, ^53^, ^54^, ^55^, ^56^ Such synthetic carbon materials are still interesting for some applications. As anode material for high‐rate applications, especially hard carbons composed of graphene‐like carbon layers with hierarchically structured pores, rich in heteroatom doping, are promising.^57^, ^58^, ^59^, ^60^, ^61^ Figure 2 shows the structure of such carbon materials. Furthermore, although graphite can only be used as an intercalation material under certain circumstances in sodium‐ion batteries, owing to the weak substrate binding energy,^62^, ^63^, ^64^ heteroatom doped hard carbons have successfully been applied. Likewise, biomass‐based porous carbon materials may be used in cathodes of lithium–sulfur,^52^, ^53^, ^54^, ^56^, ^65^, ^66^ lithium–selenium,^67^ or lithium–oxygen batteries^53^, ^56^ to host sulfur or selenium, or to catalyze the oxygen reduction/oxygen evolution reaction (ORR/OER), respectively, as discussed in several reviews. Porous carbons not only increase conductivity in such systems but also, in the case of lithium–sulfur or lithium–selenium batteries, prevent polysulfide or polyselenide dissolution in the electrolyte to some extent, owing to the adsorption properties of carbon surfaces. Furthermore, the expansion of sulfur or selenium during cycling is restricted to the carbon pores, leading to increased battery stability.

From the viewpoint of sustainability, carbon materials derived from waste biomass are especially interesting.^24^ In recent years, carbons made from rice husks,^69^, ^70^ corn or wheat straw,^71^, ^72^ coir pith,^73^ soy bean residues (from tofu production),^74^ pistachio shells,^75^ wood chips or fibers,^76^, ^77^, ^78^ grass,^79^ pine pollen,^80^ lignin,^81^, ^82^ tannic acid,^83^ or shrimp shells,^84^ among others, have been introduced as anode materials in lithium‐ or sodium‐based batteries. Similarly, all kinds of biowaste have been carbonized and used as host materials in the cathodes of lithium–sulfur, lithium–selenium, or lithium–oxygen batteries. Within recent years, for example, carbons made from waste materials such as fruit stones^85^, ^86^, ^87^ or peels,^88^, ^89^, ^90^, ^91^, ^92^, ^93^, ^94^, ^95^ algae,^96^, ^97^ nutshells,^98^, ^99^, ^100^ soybean hulls,^101^, ^102^ grain waste,^103^, ^104^, ^105^, ^106^ other plant waste,^107^, ^108^, ^109^, ^110^, ^111^, ^112^, ^113^ saw dust,^114^ and lignin^115^, ^116^, ^117^ have been described in this regard.

Organic precursors to carbon materials have the advantage that heteroatoms may be incorporated, improving selected properties. For example, N‐doping increases conductivity without the need for ultrahigh‐temperature processes.^51^ Additionally, the natural structure of plant materials may be preserved during carbonization, resulting in hierarchically structured carbons even without the introduction of templates.^79^ Still, in most cases porosity is introduced by hard templating^118^ or salt melt synthesis,^119^ followed by deconstruction of templates or washing. Instead of templating, chemical activation is widely used, for example, by KOH treatment,^68^ borax treatment,^120^ or by other chemical activation agents such as KHCO_3_ or H_3_PO_4_. In this case, the process of electrode formation often includes a precarbonization step at moderate to high temperature (ca. 300–800 °C), followed by introduction of the activation agent and etching at elevated temperature in the range of 700–900 °C. Additionally, porosity may be increased by using a template‐free approach such as puffing, that is, by compression (ca. 1 MPa) and instantaneous release of pressure at elevated temperatures (200–300 °C).^121^ This process however does not lead to sufficiently small pores, so further porogens are needed in addition. Finally, physical activation using water vapor and CO_2_ may be used to introduce porous structures in biomass‐derived carbon materials.^122^, ^123^, ^124^, ^125^

Besides being used as intercalation or host materials, carbons are omnipresent in lithium‐ion battery anodes and cathodes to support charge transfer from the active material to the current collector. These additives often also contribute to significant charge storage,^126^, ^127^, ^128^ which may falsify reported specific capacities if those are referred to the mass of active material only. As such, conductive carbons are usually oil‐based (for example, carbon Super P is made from partial oxidation of petrochemical precursors and hence not from regrown bioresources^128^), and are not discussed herein. However, conductive carbon additives may also be synthesized from renewable biomass, for example, from sawdust, tannic acid, or polysaccharides.^129^, ^130^ Such biomass‐based conductive carbons in the form of porous carbon particles of controlled size were synthesized with nitrogen contents of up to 4 % and surface areas of up to 400 m^2^ g^−1^ and successfully applied as conductive carbon additives in anode materials for lithium‐ion batteries.^129^ Importantly, the morphology and porosity play major roles in determining the final capacity, with mesoporous fibrous systems facilitating charge storage.^130^

#### 2.1.1 Anodes for lithium or sodium‐based batteries

Carbonization processes usually require high temperatures significantly above 500 °C as carbonization of biomass is incomplete at or below 500 °C. Such conditions are the main reason for the high cost of synthesized hard carbon when compared to natural graphite. A truly sustainable process would require not only sustainable starting material (waste biomass, not food‐based) but also benign process conditions with the carbonization temperature being as low and the time as short as possible. In this regard, for working at lower temperatures and consequently realizing more benign process conditions, hydrothermal carbonization of renewable carbohydrates was described.^131^ Temperatures in the range of 200 °C under self‐generated high pressure and aqueous environment enable more sustainable process conditions. Importantly, besides carbohydrates, also actual waste biomass was successfully converted into hydrothermally carbonized material.^132^ However, the resulting material obtained under such comparably benign conditions is still approximately only made up by 70 % from carbon atoms and especially has a high amount of carboxylic groups on the surface. To be used as an anode material in lithium or sodium batteries, consequently a further carbonization step at significantly higher temperatures is still required.^133^ Furthermore, chemical activation, porogens, or templates are necessary to increase the porosity.^134^

Of all biomass‐based carbon materials for anode applications, some recent examples that require comparably benign process conditions shall be introduced next. Carbonization temperatures as low as 700 °C were used to make anode materials for lithium‐ion batteries. Using wheat straw,^68^ olive stones,^135^ chitosan,^119^ which is gained from chemical treatment of chitin (e.g., from the exoskeleton of crustaceans), ramie fibers and corncobs,^136^ egg yolk,^120^ human hair,^137^ or glucose,^118^ different researchers obtained carbons with good lithium storage properties as anode material in half cell experiments. Heteroatoms are often incorporated from the bioresource itself. Capacities are rather high, for example in the range of 1300 mAh g^−1^ at slow charge–discharge rates of 0.037 A g^−1^, and drop to values in the range of 200 mAh g^−1^ upon fast charge–discharge of 37 A g^−1^.^68^ Often, no clear voltage plateau can be observed. Together, this behavior indicates a large contribution of lithium deposition in the micropores at slow charge–discharge rates.

At even lower carbonization temperatures of 600 °C, Lim et al. carbonized wheat flour. At such low temperatures, the final material contains a high concentration of nonhydrocarbon impurities. The material could still demonstrate capacities of almost 400 mAh g^−1^ at 0.1 C and 220 mAh g^−1^ at 1 C, which is rather high given that no acid or base washing of the raw material was performed and no porogen was used.^138^ The cycling stability was higher than for most waste biomass‐based materials. The authors explained this behavior with the good morphological control when using well defined starch‐based precursors in contrast to waste biomass‐derive carbons. Still, using flour as a carbon source inheres using food biomass for carbonization and therefore decreases the degree of sustainability of this process.

Li et al. carbonized corn straw at temperatures as low as 550 °C. After activation with KOH, a mesoporous carbon material resulted with capacities in the range of 500 mAh g^−1^ at 0.2 C when used as an anode material in a lithium battery setup.^72^ Despite a high irreversible capacity in the first cycle, which was attributed to formation of an SEI film on the porous carbon surface, rather stable cycling was demonstrated.

**Figure 3:**
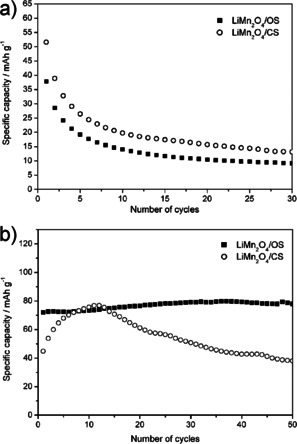
Electrochemical performance of olive stone (OS)‐ and cherry stone (CS)‐derived carbons as anode materials in a full cell setup with LiMn_2_O_4_.cathodes. Reproduced with permission from ref. 135. Copyright 2011 by Wiley‐VCH.

Finally, through carbonization of cherry stones at 500 °C with KOH or ZnCl_2_ activation, Arrebola et al. generated carbonaceous materials with a carbon content of up to 86 %, a significant oxygen content, and comparably high hydrogen content (around 2.5 %).^139^ They proposed that the low carbonization temperature actually increases the performance if the first cycle charge is limited (to prevent a high irreversible capacity). At 0.2 C, the authors achieved almost 350 mAh g^−1^ and more than 250 mAh g^−1^ at 1 C. However, when used in a full cell setup, the performance was very poor (around 10 mAh g^−1^ and, after optimization with 5 initial cycles in a half‐cell setup, still only about half of the theoretical value; Figure 3).^135^ This observation demonstrates that it is not simple to exchange the graphite used in state‐of‐the‐art lithium‐ion batteries with a bioderived carbon. Actually, there is barely any work on sustainable carbon materials in full cells, meaning that the quest for a good carbonaceous anode material from biomass for true battery applications has just begun.

#### 2.1.2 Cathodes for lithium–sulfur, lithium–selenium, and lithium–oxygen batteries

**Figure 4:**
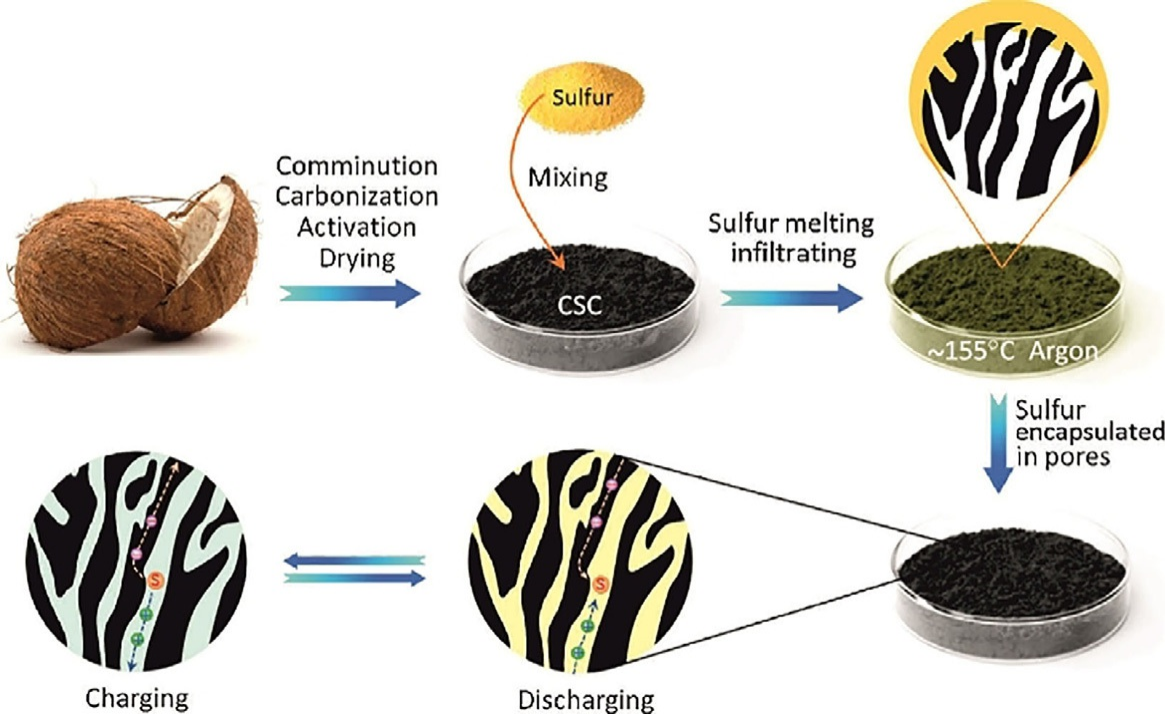
Schematic representation of the formation of sulfur‐filled carbon materials to be used as cathodes in lithium–sulfur batteries. Reproduced with permission from ref. 91. Copyright 2017 by the American Chemical Society.

In lithium–sulfur batteries, carbons may serve as host for sulfur. Hence, they need to be impregnated with sulfur by melting elemental sulfur in the presence of the carbon or deposition from solution, for example, in carbon disulfide, or disproportionation of sulfur from thiosulfate in acidic solution (Figure 4). Therefore, carbons with high surface areas usually find application. The carbon–sulfur composites usually contain 50–75 % sulfur and are processed together with another carbon of great conductivity, such as carbon super P, and a binder, such as polyvinylidene fluoride (PVDF), resulting in an approximate sulfur content in the total electrode of 40–60 %. Capacities typically range from 800 to 1200 mAh g^−1^ (referred to the mass of sulfur) at slow cycling speed and lose 30–50 % of the initial value within the first 100 cycles. This loss of capacity might be caused by polysulfide dissolution even though the sulfur is rather confined within the pores.

Despite significantly lower natural abundance, selenium has been presented as an alternative to sulfur in lithium–chalcogenide batteries because of similarly high volumetric capacity and 20‐fold higher conductivity.^140^ Because of the latter, carbon as a conductive support is not mandatory, however, it helps to prevent dissolution and volume expansion issues.^67^ Impregnation of biomass‐based carbons with selenium proceeds in a similar manner to impregnation with sulfur, that is, usually by mixing both components and heating them above the melting point of selenium.^112^, ^115^ Specific capacities are lower than in lithium–sulfur batteries (typically 500–700 mAh g_selenium_^−1^, decaying by 20–50 % within the first 100 cycles), owing to the lower theoretical capacity.

Carbons to be used in lithium–oxygen batteries or in general as catalysts for the oxygen reduction/oxygen evolution reaction require heteroatom doping to catalyze the reactions.^141^ Nitrogen doping, in the form of pyridinic and graphitic nitrogen, is especially beneficial owing to the weakening effect on the O−O bond and enhanced electron transfer, respectively.^53^ Carbons made from biomass often naturally benefit from nitrogen doping due to the presence of nitrogen in many bioresources^142^, ^143^ and are thus especially appealing. In cases where N‐doping from the carbon precursor is not sufficient, other nitrogen containing precursors like melamine, which can be synthesized from sustainable resources, may be added before carbonization.^96^, ^97^ To decrease the overpotential and increase the efficiency of the ORR/OER, biomass‐based carbons may also be impregnated with metal ions or particles.^95^, ^97^, ^144^ The remaining synthesis steps are similar to the above‐described processes.

**Figure 5:**
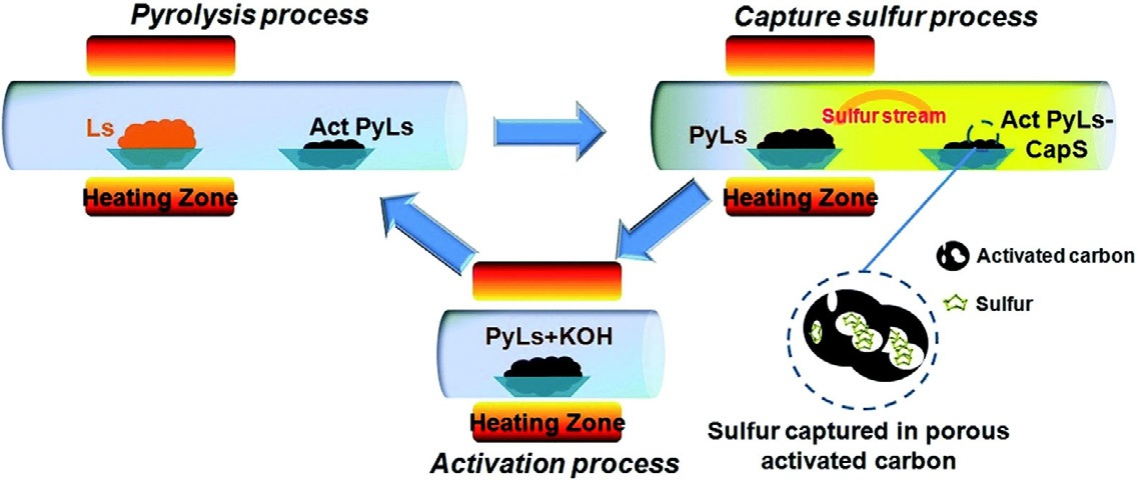
Schematic representation of the fabrication process of sulfur‐loaded carbons using lignosulfonate as carbon precursor. Reproduced with permission from ref. 117. Copyright 2018 by the Royal Society of Chemistry.

Besides the standard procedures for cathode formation introduced above, some innovative approaches have been developed. For example, the group of Simmons worked on lignosulfonate‐based carbons for lithium–sulfur batteries.^116^, ^117^ Lignosulfonate is a low value byproduct of the paper industry rich in sulfur in the form of sulfonic acid groups. In a circular pyrolysis approach, sulfur that is lost during carbonization of lignosulfonate is incorporated into a second batch of previously activated and carbonized lignosulfonate (Figure 5). Consequently, addition of less sulfur to the carbon material to be used in lithium–sulfur batteries is necessary.

Rice husks are also interesting as they naturally contain large amounts of silica. Consequently, no further porogen needs to be added (although it admittedly still increases the performance^104^ or enables lower processing temperatures^105^) and only one carbonization step, followed by washing of the silica is necessary to achieve porous carbons.^103^ Li et al. reported an innovative approach for the introduction of pores into biomass‐based carbon materials for lithium–sulfur batteries.^93^ The authors fermented banana peels with yeast and thereby introduced micro‐, meso‐, and macropores by aerobic respiration and anaerobic breathing of yeast. During carbonization, the yeast also led to introduction of nitrogen into the carbon material, enhancing the electrochemical properties of the carbon/sulfur cathode material.

Nevertheless, as mentioned above, capacity in lithium–sulfur batteries, even with (biomass‐based) carbon cathodes, undergoes severe fading within the first 100 cycles owing to polysulfide dissolution. Additives such as metal oxides or metal sulfides might be beneficial to partly suppress this process.^145^ For example, Moreno et al. reported that olive stone‐derived carbons that contain TiO_2_ show significantly enhanced capacity retention and improved rate capability, as TiO_2_ prevents polysulfide diffusion into the electrolyte.^86^ However, despite such promising results, no significant commercial applications have been described to date. Similarly, the overpotential in lithium–oxygen batteries is rather high, and the batteries usually only work in pure oxygen atmosphere. Reversible ORR/OER in lithium–oxygen batteries has not yet been achieved in prototype cells with high specific capacities.^146^ Consequently, improvements are necessary before they will find widespread commercial application.

### 2.2 Noncarbonized biomass‐based organic electrode materials

Redox‐active biomolecules may serve as promising electrode materials in batteries themselves, without any carbonization step. The redox potential in combination with the counter electrode determines whether a biomolecule may rather be suited as the anode or cathode. Sustainable, noncarbonized, biomass‐based materials may be advantageous, especially in anodes of sodium‐ion batteries in which graphite cannot readily be used or in cathode materials of lithium‐ion batteries, where carbons are conventionally only used as a conductive additive. However, as redox‐active molecules in nature are often soluble in polar solvents, such as electrolytes, using them as active materials in organic batteries often causes stability problems as the active material is dissolved in the electrolyte. Dissolution may be prevented by using solid electrolytes^147^ or a high amount of mesoporous carbon additive,^148^ which however decreases the conductivity or charge density. Besides utilization in redox flow batteries, redox‐active bioderived molecules may consequently benefit from insolubilization, for example, by salt formation or incorporation in polymers.

#### 2.2.1 Carboxylates

One class of substances that has been thoroughly investigated for organic anode materials is carboxylates. Besides often exhibiting rather low redox potentials, many carboxylates are available from renewable biomass.

##### 2.2.1.1 Terephthalates

**Figure 6:**
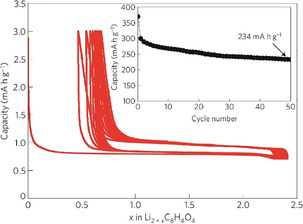
Galvanostatic charge–discharge experiments for lithium terephthalate‐based anodes at slow charge–discharge rate of 1 Li^+^ per 10 h. Reproduced with permission from ref. 149. Copyright 2009 by SpringerNature.

The first prominent example of an organic salt as an electrode material was in 2008, when Armand et al. reported conjugated dicarboxylates lithium terephthalate and lithium muconate as anode material in sustainable batteries.^149^ Although in usual lithium‐ion batteries, common battery electrolytes start to decompose at low temperatures in the range of 50 °C and lead to dangerous combustion products and battery fires,^150^ the same electrolytes are more stable when used together with these sustainable electrodes.^149^ Electrodes based on terephthalates are especially promising, as they can not only be synthesized from *p*‐xylene but also from recycled PET plastic and are thus not only safer but also significantly greener than common anode materials.^126^ Thanks to increasing worldwide environmental awareness, furthermore synthetic routes for terephthalates from bioresources have been developed and applied.^151^, ^152^, ^153^ Terephthalate‐based anode materials hence surely qualify as sustainable electrodes as they are also potentially bioderived materials. Figure 6 exemplarily illustrates the performance of lithium terephthalate‐based anodes. Within the last ten years, many researchers followed up on Armand's initial research and described the use of terephthalate‐based anodes in lithium‐ion,^154^, ^155^, ^156^, ^157^, ^158^, ^159^, ^160^, ^161^, ^162^, ^163^, ^164^, ^165^ sodium‐ion,^126^, ^159^, ^166^, ^167^, ^168^, ^169^, ^170^, ^171^, ^172^, ^173^, ^174^ or potassium‐ion^127^, ^165^, ^175^, ^176^, ^177^ batteries.

At potentials around 0.8 V vs. Li^+^/Li, 0.4 V vs. Na^+^/Na, and 0.6 V vs. K^+^/K, di(alkali metal) terephthalates can reversibly insert up to 2 atoms of the respective ion and deliver theoretical reversible capacities of 301 mAh g^−1^ (dilithium terephthalate), 255 mAh g^−1^ (disodium terephthalate), and 222 mAh g^−1^ (dipotassium terephthalate). Upon introducing substituents to the aromatic ring, both the potential and the capacity can further be tuned.^126^, ^162^, ^163^, ^164^ Practically achievable capacities are initially often close to theoretical values and undergo slow fading, for example to around 230 mAh g^−1^ (at 15 mA g^−1^ after 50 cycles) in a lithium half‐cell setup^149^ or around 165 mAh g^−1^ (at 0.1 C after 50 cycles) in a sodium half‐cell setup.^173^

Some researchers have reported even higher capacities than the theoretical capacities of the di(alkali metal) terephthalates themselves.^126^, ^127^, ^160^, ^161^, ^167^, ^174^, ^178^ Such improbable high capacities may be explained if specific capacity is calculated relative to the mass of the active material only, although additives contribute to additional charge storage. Prominently, carbon additives may contribute to additional capacitive charge storage to some extent (e.g., carbon black Super P), and deposition of the alkali metal in porous morphologies results in charge storage as discussed above. Furthermore, additional metal ions may be incorporated at the cost of losing aromaticity.^179^ Further reasons may be discharging to potentials at which the structure is decomposed and more than the two reversibly cyclable lithium, sodium, or potassium ions contribute to charge storage. Referring specific capacities to the total electrode mass instead of the mass of active material would to some extent help to better compare the performance in different studies. Even if thoroughly discussed, such results unfortunately set the bar for desired capacities higher than rationally achievable by the terephthalate alone and should be considered carefully by other researchers and reviewers. Of course, such questionable reports are not limited to terephthalate‐based anodes but occur for all kinds of reported electrodes.

Importantly, even in a full‐cell setup with organic cathode, terephthalate‐based anodes may be used. The reported energy density is in the range of 130 Wh kg^−1^ for lithium‐ion batteries^155^ and 65 Wh kg^−1^ for sodium‐ion batteries,^168^ which brings terephthalate anodes one step closer to practical application compared to biomass‐based carbon anodes.

**Figure 7:**
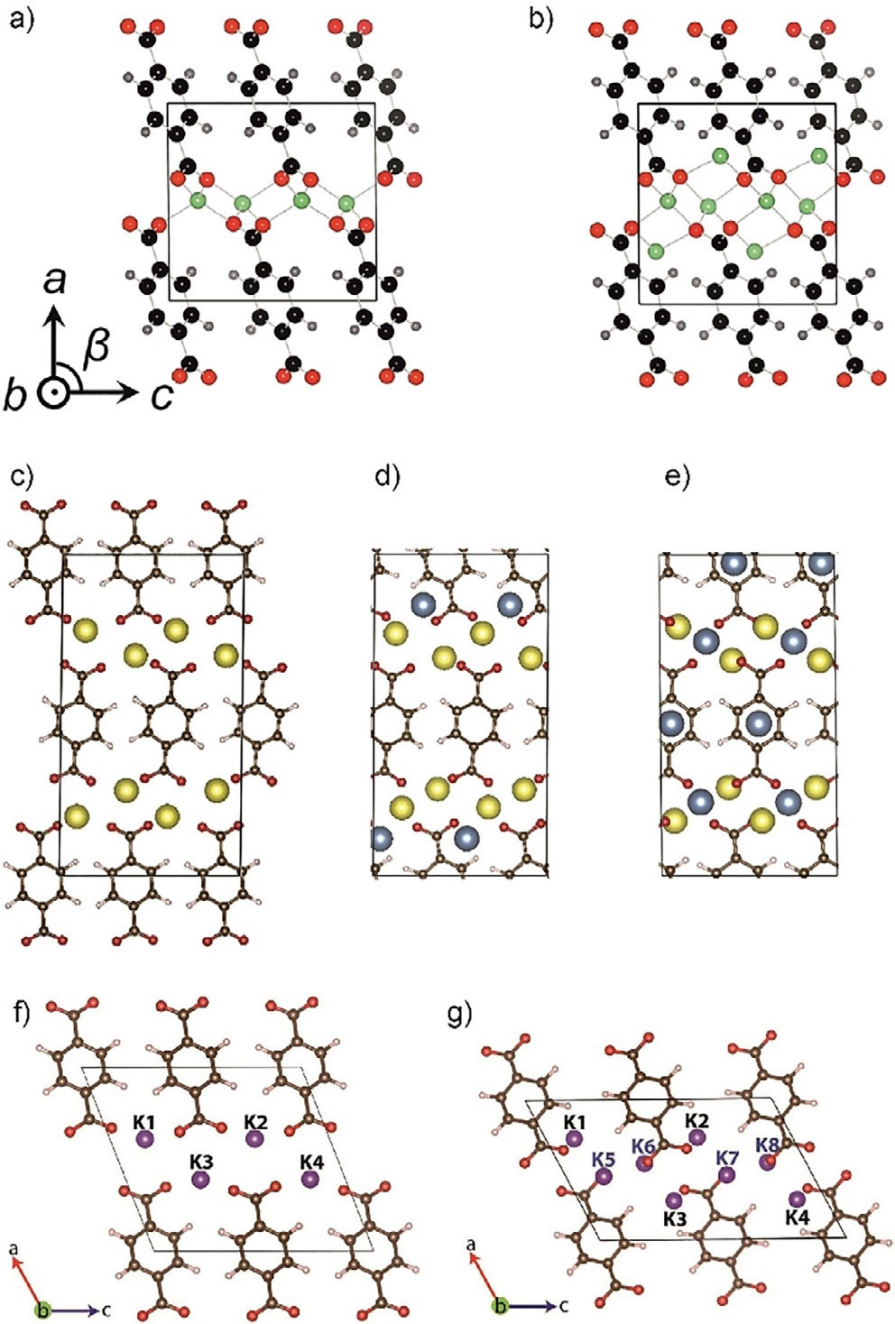
Incorporation of alkali metal ions in di(alkali metal) terephthalates: a, b) Dilithium terephthalate before (a) and after (b) incorporation of two additional lithium ions. Reproduced with permission from ref. 154. Copyright 2012 by AIP Publishing. c–e) Disodium terephthalate before (c) and after incorporation of one (d) or two (e) additional sodium ions. Reproduced with permission from ref. 170. Copyright 2016 by Elsevier. f, g) Dipotassium terephthalate before (f) and after (g) incorporation of two additional potassium ions. Reproduced with permission from ref. 177. Copyright 2017 by Elsevier.

In di(alkali metal) terephthalates, a layered structure with metal ion conducting channels is pertinent also upon additional ion insertion (Figure 7). Metal–organic networks consisting of positively charged alkali metals coordinated by terephthalate anions with two complexing groups are present in the structure.^126^, ^149^ Consequently, despite being based on a small molecule, dilithium (or disodium) terephthalate‐based anodes have reasonable stability and do not readily dissolve in some electrolytes.

Remarkably, the structure of fully sodiated disodium terephthalate is different from that of fully lithiated dilithium terephthalate or fully potassiated dipotassium terephthalate. Whereas in the lithium or potassium salts, both inserted lithium or potassium atoms are additionally bound to oxygen from the carboxylate groups,^154^, ^177^ only one of the two inserted sodium atoms per terephthalate unit (first insertion step) is complexed by oxygen atoms from the carboxylate groups. The second inserted sodium atom is bound to hexagonal sites between the aromatic rings (Figure 7).^170^

Upon charging or discharging, lithium ions move between the layers. Limited ion mobility and conductivity can be overcome by making high surface‐area materials, for example, nanosheet‐like morphologies^155^, ^169^ or nanocrystals.^173^, ^174^ For increasing conductivity, carbon materials such as graphite^157^ are commonly added, which may contribute to additional capacity upon ion insertion into the carbon material.^126^ To establish better conductivity and high surface area at the same time, carbon nanotubes^161^ or freeze‐dried/sintered or electrochemically reduced graphene oxide may also be added.^171^, ^172^ As expected, especially at high discharging rates, small particle sizes lead to increased capacity compared to larger particle sizes. Interestingly, as an alternative to adding conductive additives, conductive additives may in situ be formed for example when using silver terephthalate as starting material (in situ formation of silver nanoparticles).^159^

For increasing stability, other metal‐organic frameworks (MOFs) employing terephthalate ligands have been introduced, for example based on calcium terephthalate, however often with reduced capacity.^157^, ^158^ Higher capacity terephthalate MOFs could be realized at the cost of the incorporation of unsustainable metals, such as cobalt.^156^, ^175^, ^176^ Importantly, in such MOFs, lithium or sodium ions are not just incorporated at the aforementioned positions but rather in the porous structure, enabling significantly higher capacities than in conventional terephthalate‐based anodes.

Another possibility to increase the stability of redox functionalities is by incorporating them in a polymer. In this regard, polymers with pendant terephthalate groups have been synthesized and tested in lithium and sodium half‐cell experiments.^180^, ^181^ Indeed, the stability of the resulting anodes is significantly improved compared to terephthalate small molecule‐based anodes. The pitfall, however, is that the monomers are not readily available from bioresources.

##### 2.2.1.2 Other conjugated multicarboxylates

Terephthalates are the dominant species in the research area of biomass‐based organic anode materials (noncarbonized). Still, some similar conjugated carboxylates have also been described. For example, 2,5‐pyridinedicarboxylic acid is structurally similar to terephthalic acid with the difference that the aromatic ring is pyridine in this case. Dipotassium 2,5‐pyridinedicarboxylate has been investigated as an anode material in potassium ion half‐cell experiments and resulted in similar capacities to dipotassium terephthalate.^177^ However, given that it is not readily available from biomass, there is no clear advantage in using 2,5‐pyridinedicarboxylic acid instead of terephthalic acid.

A means to boost the capacity of terephthalates is to substitute some or all oxygen in the carboxylic groups by sulfur, which facilitates charge delocalization and accessible capacity. In fact, the resulting thiocarboxylates reach very high capacities of more than 550 mAh g^−1^ at a current density of 50 mAh g^−1^ in a sodium battery setup.^182^ This boost in capacity, however comes at the price that, although it is structurally similar to terephthalic acid, thioterephthalic acid is not readily available from bioresources.

Interestingly, a conjugated system between carboxylates seems not to be crucial for energy storage. 1,4‐cyclohexanedicarboxylic acid with a cyclohexane ring instead of a benzene ring between the carboxylic acids may also be used in sodium‐ion battery anodes. Whereas delocalization of electrons helps to stabilize radicals that form as intermediates in charge–discharge processes, 1,4‐cyclohexanedicarboxylic acid is also remarkably stable with a capacity in the range of 150 mAh g^−1^ after 100 cycles at 100 mA g^−1^.^183^

**Figure 8:**
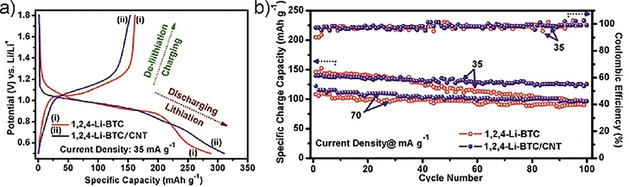
Electrochemical behavior of 1,2,4‐benzenetricarboxylic acid lithium salt (1,2,4‐Li‐BTC) without and with added carbon nanotubes for stabilization at slow cycling rates as indicated. a) First charge–discharge cycle. b) Long‐term cycling behavior within 100 cycles. Reproduced with permission from ref. 184. Copyright 2017 by Elsevier.

Instead of these species, which are similar to terephthalates but not available from bioresources, bioavailable similar species may be used. For example, upon oxidizing rosin, which is obtained by distilling pine resins, trimellitic acid can be obtained. In this 1,2,4‐benzenetricarboxylic acid, carboxylate groups are conjugated, as in terephthalic acid, and Maiti et al. recently described its use as anode material in lithium‐ion batteries.^184^ The material exhibits slightly lower capacities than terephthalate‐based anodes (cf. Figure 6) in the range of 150 mAh g^−1^ but is comparatively more stable in composites with carbon nanotubes. Figure 8 illustrates its electrochemical behavior at slow cycling rate.

Croconic acid is not readily available from biomass but can easily be synthesized from charcoal and potassium carbonate. Its disodium salt has been described as a possible anode material for sodium‐ion batteries.^185^ In the pristine form, it suffers from fast decay of capacity through pulverization of particles and loss of contact during sodiation‐induced volume change. This decay can be reduced to some extent by using smaller particles or wrapping them with graphene oxide, leading to similar capacities to the other discussed materials (ca. 150 mAh g^−1^ after 50 cycles at 20 mA g^−1^).

To enhance the charge delocalization in conjugated dicarboxylates and to attain improved capacities, rate performance, and cycling stability,^22^ anodes based on dicarboxylates with extended conjugated π‐systems, such as 1,4‐benzenediacrylate,^186^, ^187^ 2,6‐naphthalenedicarboxylate,^166^, ^188^, ^189^, ^190^, ^191^, ^192^ 1,4,5,8‐naphthalenetetracarboxylate,^193^ 3,4,9,10‐perylenetetracarboxylate,^194^, ^195^, ^196^ 4,4′‐biphenyldicarboxylate,^197^ 3,3′,4,4′‐biphenyltetracarboxylate,^198^ 4,4′‐stilbenedicarboxylate,^199^ or 4,4′‐tolanedicarboxylate,^200^ have been developed. Although some of these polycyclic aromatic molecules are available in trace amounts from renewable bioresources, obtaining them from renewable biomass is not feasible and thus electrodes based on polycyclic aromatic di‐ or multicarboxylates or benzenediacrylate shall not be discussed herein. Instead, the reader is referred to a recent review by Häupler et al.^22^

Other polycyclic aromatic systems with carboxylate groups are indeed readily available in nature. One prominent and widely distributed material is humic acid. It is rich in carboxylic groups and, despite not providing clear plateau‐like charge–discharge behavior, has been successfully used in anodes for lithium or sodium half cells,^201^ and even in full organic cells with cathodes based on emodin, which is a natural anthraquinone.^202^ Such natural quinones as redox‐active materials in general are discussed next.

#### 2.2.2 Quinones and similar carbonyls

Quinones are especially appealing electrode materials, as several hydroquinones or quinones occur naturally in the environment, and their redox potentials are usually in the range of 1.5–3.5 V vs. Li^+^/Li. Several reviews specifically focus on all kinds of quinones in battery cathodes.^27^, ^33^, ^36^ As the redox potential, depending on the substituents, can be tuned over a rather large range, quinones may find application both in anodes and in cathodes.

In several especially interesting recent reviews, Wang et al. and Lee et al. summarized the use of natural or nature‐inspired quinones and flavins in electrochemical energy storage devices, focusing on small molecules.^32^, ^38^, ^203^ Studying these reviews is highly recommended. For a bigger picture, in addition to small molecules, I provide herein a particular focus on polymeric biomass‐based electrodes and immobilized small molecules in biomass‐based electrodes.

##### 2.2.2.1 Benzoquinones/catechols

Several natural compounds contain catechol functionalities, which may be reversibly oxidized to form *o*‐benzoquinones. For example, some flavonoids, such as catechin and epicatechin, which build up procyanidins and which are found in many fruits,^204^ or, amongst others, the anthocyanidins cyanidin, cyanin, chrysanthemin, antirrhinin, delphinidin, or europinidin behave accordingly.^205^ Whereas, even without immobilization, such natural small molecule catechols and catechols incorporated in a biopolymer may find application as electrolytes in organic redox flow batteries, owing to their reversible redox behavior,^206^, ^207^ most other charge storage applications require fixation on a support or incorporation in a polymer backbone. Approaches in this regard together with applications have recently been summarized in an excellent review by Patil et al.^208^ Importantly, catechols strongly complex multivalent ions and thus may also be employed in some post‐lithium‐ion‐batteries.^209^

**Figure 9:**
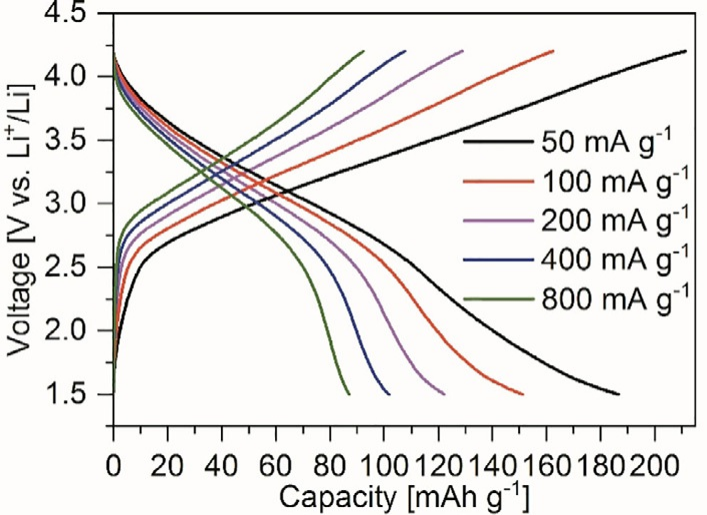
Galvanostatic charge–discharge behavior of cathodes featuring polydopamine‐*co*‐pyrrole polymer as active material, clearly illustrating the mixed battery‐like and capacitor‐like behavior frequently observed in organic electrodes. Reproduced with permission from ref. 217. Copyright 2018 by Elsevier.

One catechol‐containing small molecule that has been extensively studied within recent years is dopamine.^210^ Supported by carbon nanotubes, which contribute to capacitive charge storage, self‐polymerization enabled its use as a cathode material in lithium‐ or sodium‐ion batteries.^211^ Additionally, physical properties such as adhesion,^212^, ^213^, ^214^ conductivity,^212^, ^213^, ^214^, ^215^ and biocompatibility^216^ of copolymers with pyrrole have been extensively studied. Liedel et al. recently also investigated the application of such copolymers as cathode materials for lithium‐based batteries.^217^ As is obvious from the galvanostatic charge–discharge behavior (Figure 9), which features neither clear plateau‐like nor triangular behavior, battery‐like charge storage through distinct redox reactions of quinone functionalities was supported by the pseudocapacitance of the conjugated backbone. Such synergistic effects of different charge storage mechanisms are promising for future bioorganic energy storage devices.

**Scheme 1:**
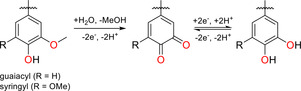
Redox behavior of guaiacyl‐ or syringyl‐containing biomolecules in aqueous electrolytes. Adapted from ref. 219, published by The Royal Society of Chemistry.

This approach of combining conjugated polymers with biopolymers that contain specific precursors for benzoquinones has prominently been used in case of lignin‐based charge storage devices. Although lignin does not contain redox‐active functionalities itself, oxidation (e.g., electrochemically in aqueous media) results in the transformation of guaiacol and syringol groups into (substituted) 1,2‐benzoquinones (Scheme 1).^218^

First reported by Milczarek and Inganäs,^220^ such electrodes have been summarized in several reviews recently.^29^, ^221^, ^222^ In particular, combinations with conductive polymers such as polypyrrole,^220^, ^223^, ^224^, ^225^, ^226^, ^227^, ^228^, ^229^, ^230^ polyaniline,^229^, ^231^ or poly(3,4‐ethylenedioxythiophene) (PEDOT)^229^, ^232^, ^233^, ^234^, ^235^, ^236^, ^237^ have been described. Addition of polysaccharides for stability and binder purposes resulted in particularly sustainable electrodes.^225^, ^234^, ^237^ Importantly, whereas guaiacol and syringol groups in lignin are oxidized during electropolymerization of aniline and EDOT in the presence of lignin, post‐synthesis oxidation is necessary in the case of pyrrole polymerization in the presence of lignin.^229^ The final redox‐active materials were almost exclusively used in aqueous electrolytes, with rare exceptions, for example, when lignin was used as a dopant for PEDOT.^235^ Such doping is facilitated when lignosulfonates are used as these negatively charged polyelectrolytes may exchange counterions with doped PEDOT, resulting in enhanced charge storage performance by more than 60 % through reversible redox reactions.^236^ Notably, lignin‐derived phenolic acids may also be used as dopants for polymers, resulting in similar enhancement of the charge storage performance.^238^ In this case, the choice of lignin monomer greatly influences the performance of the final electrodes, with guaiacol units enabling higher charge storage than syringol units.^239^

As discussed above, carbonized lignin may be used as an anode material in sustainable batteries.^81^, ^82^ Redox‐active groups may be retained if pyrolysis of lignin/polypyrrole composites is performed under mild conditions, resulting in a carbonaceous material with distinct, lignin‐derived, redox functionalities.^230^

Furthermore, instead of conjugated polymers different carbon materials may be added to lignin not only to promote conductivity but also to provide the possibility of synergistic charge storage on carbon surfaces and in catechol groups. Different carbonaceous species, such as graphene,^240^, ^241^, ^242^ carbon nanotubes,^243^ or other high‐surface area carbons^244^, ^245^ have been presented in this regard. Besides providing conductivity, intelligent design of the carbonaceous material may increase stability by preventing dissolution of the active material.^242^ In terms of sustainability, the use of biomass‐based carbons is especially appealing, and resulting hybrid materials showed reasonable performance with a clear combination of battery‐like and capacitor‐like charge storage mechanism.^244^ For enhanced sustainability of lignin‐carbon hybrid materials, fluorinated binders may be omitted, especially if lignin is crosslinked to promote stability.^245^ Importantly, the combination of redox‐active biopolymers and carbon materials results in synergistic enhancement of capacitive and battery‐like charge storage and is not merely a combination of the two. Such synergistic effects may be explained by changes in the carbon's hydrophilicity upon incorporation of biopolymers, facilitating interactions with polar electrolytes and thus resulting in enhanced double layer formation,^246^ similar to other charge storage materials.^247^ Furthermore, the choice of carbon plays a major role for performance. Not only the surface chemistry, which facilitates interactions with the biopolymer, influences charge storage, but also especially the surface area and porosity determine the electrochemical behavior of such hybrid materials.^248^

Another class of abundant naturally occurring polyphenols is tannins, which are widespread, for example, in wood bark and often bear a high density of catechol‐like functionalities. Similar to lignin, some tannins may be used in combination with conjugated polymers^249^ or carbons^250^, ^251^, ^252^, ^253^ for charge storage applications. Moreover, in combination with biomass‐based carbons, the omittance of fluorinated binders or hazardous solvents is especially appealing for fabricating sustainable electrodes.^253^

Although lignin and tannic acid show great potential for sustainable biogenic batteries, owing to their high natural abundances, their densities of possible redox‐active units are rather low. For example, in the case of lignin, most guaiacol and syringol building blocks are etherified or esterified in the complex network structures. Consequently, lignin‐derived small molecules bearing the same functionalities as lignin may be more appealing for applications. Vanillin, which can be synthesized on an industrial scale from lignin, may be the most promising candidate in this regard. Liedel et al. recently immobilized it on a chitosan backbone, whereby it demonstrated general suitability as a cathode material.^219^ The authors furthermore polymerized it without chitosan and built a crosslinked redox‐active network with applicability as a cathode material in lithium‐ion batteries.^254^

**Figure 10:**
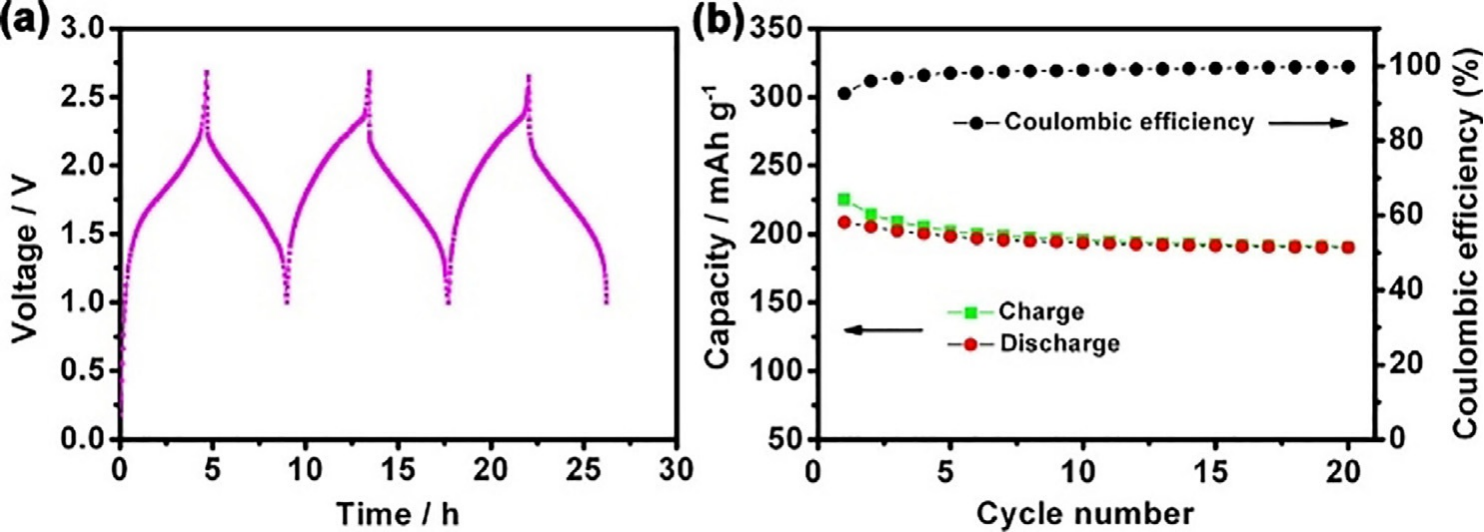
Galvanostatic charge–discharge behavior (a) and cyclability (b) of lithium‐ion batteries featuring 2,5‐dihydroxyterephthalic acid lithium salt as active material in both electrodes. Reproduced with permission from ref. 155. Copyright 2013 by the American Chemical Society.

Finally, 2,5‐dihydroxyterephthalic acid may be used both as an anode (carboxylate groups) and as a cathode (quinone groups) material in sustainable batteries, as introduced above. It may be formed by carboxylation of hydroquinone and subsequent lithiation or sodiation and contains both quinone and carboxylate moieties. With a cell voltage of approximately 1.8 V and energy densities of about 130 and 65 Wh kg^−1^ for lithium‐ and sodium‐ion batteries, respectively,^155^, ^168^ it is rather appealing for future applications. Figure 10 shows the performance of respective lithium‐ion battery full cells featuring the active material in the anode and cathode.

##### 2.2.2.2 Naphthoquinones

**Figure 11:**
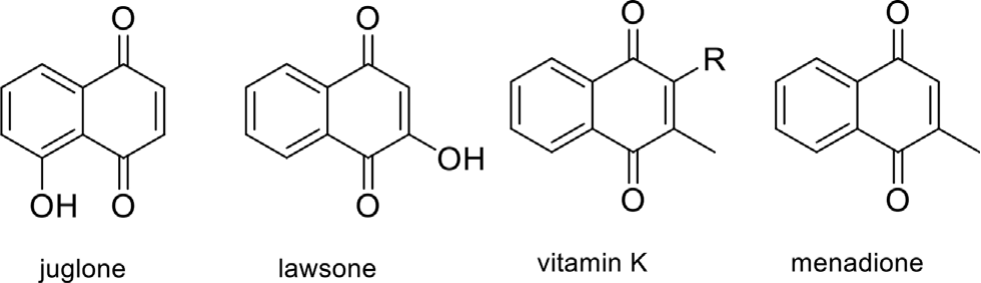
Natural naphthoquinones juglone, lawsone, and vitamin K, together with menadione, the synthetic form of vitamin K.

Juglone and lawsone, two natural dyes (Figure 11), are substituted naphthoquinones that exhibit significant redox activity.^255^, ^256^, ^257^, ^258^, ^259^, ^260^, ^261^ Recently, they have been used in a range of electrochemical applications, such as supercapacitors,^256^, ^257^, ^260^ lithium‐ion batteries,^258^, ^261^ or sodium‐ion batteries.^255^ To prevent their dissolution in the electrolyte, nanowires were formed by an antisolvent technique, that is, by recrystallization or by dropping a solution of the redox‐active biomolecule into an antisolvent in which crystals then form in high nm or low μm concentrations.^259^ As expected, smaller diameters result in better charge storage properties. Alternatively, hybrid materials with reduced graphene oxide were investigated.^255^ With reversible redox reactions at potentials in the range of 2.3 V vs. Li^+^/Li, the material might be usable either as an anode^257^ or as a cathode^258^ material, depending on the second electrode. Furthermore, redox flow batteries were developed, embracing the good solubility in the electrolyte.^262^

Vitamin K is structurally similar to juglone and lawsone and based on a naphthoquinone core (Figure 11). Instead of a hydroxy group, its structure includes a methyl group in the 3‐position and a side chain of variable length in the 2‐position. The synthetic provitamin menadione, without this side chain (Figure 11), has been presented as anode material for potassium‐ion batteries, with a surprisingly low redox potential in the range of 1 V vs. K^+^/K and hence approximately 1.2 V lower than in lawsone and juglone (other studies^263^ showed redox activity of vitamin K in the range of −0.3 V vs. Ag/AgCl).^264^ Charge storage in the range of 220 mAh g^−1^ after 100 cycles at 100 mA g^−1^ was presented as a combination of redox activity of menadione and significant contributions of graphene nanotubes used for immobilization and acetylene black used as conductive additive. As natural vitamin K has a significantly higher molecular weight, lower capacity would be expected when the biomolecule was used as an anode material instead.

##### 2.2.2.3 Anthraquinones

Polymerized anthraquinones probably make up the major share of described quinone‐based polymeric electrode materials. Unfortunately, the materials are almost exclusively available from petrochemical resources,^265^ with the most sustainable production route from anthracene, derived from coal tar and thus from a (not biomass‐based) waste material. Whereas pure anthraquinone can be found in nature in the form of the extremely rare mineral hoelite,^266^ several substituted anthraquinones have also been obtained by extracting biomass. Such biogenic anthraquinones are described here. A cathode based on emodin was already discussed above as part of a fully organic battery.^202^ To stabilize the material, a composite with carbon nanotubes was used. Interactions between the aromatic ring and the carbon hamper dissolution of the active material and consequently increase the cycling stability. In combination with polypyrrole, emodin was furthermore employed as a cathode in wearable supercapacitors.^267^

**Figure 12:**
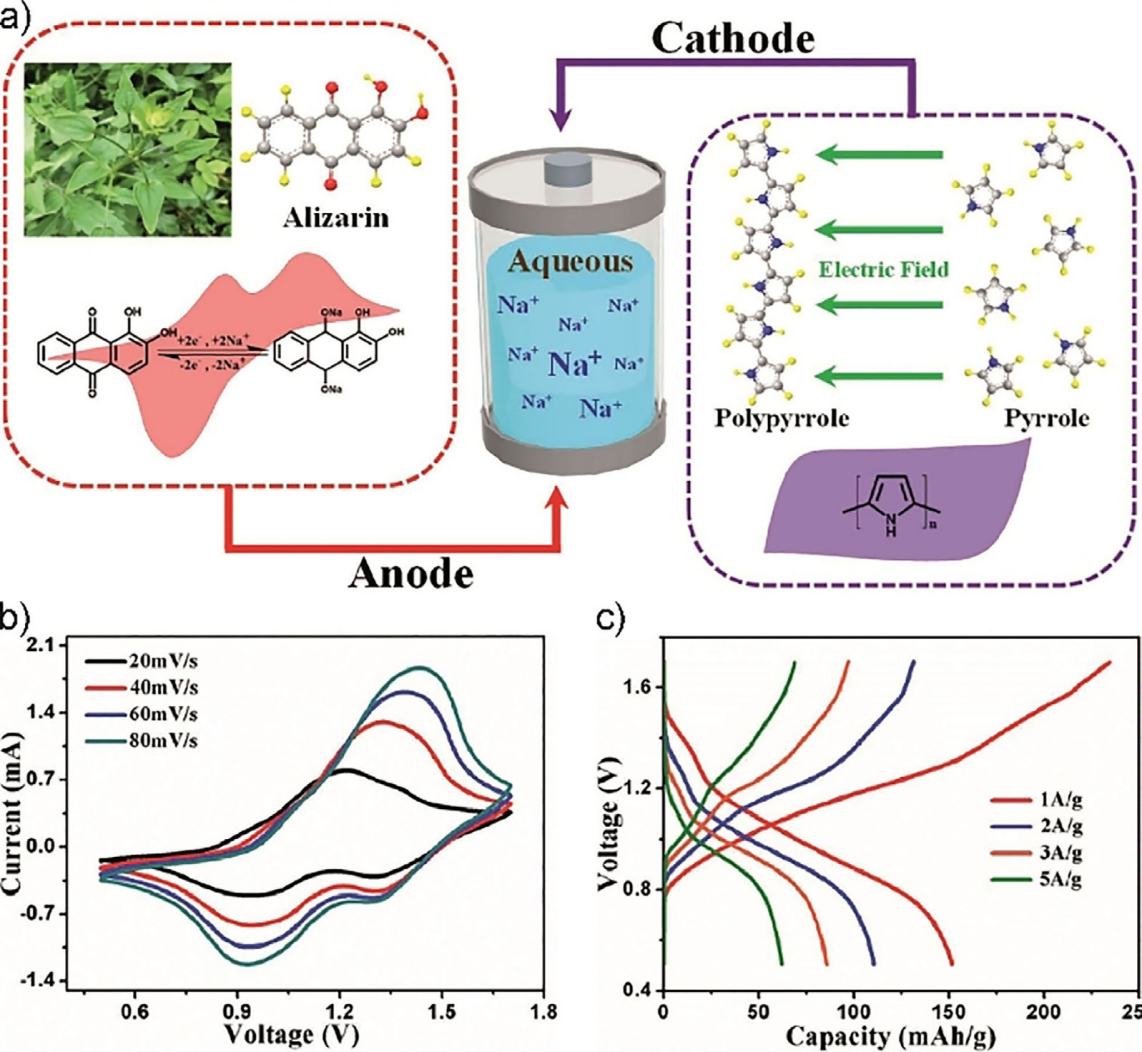
Setup of an alizarin‐based sodium‐ion battery with polypyrrole counter electrode (a) and its electrochemical performance as determined by cyclic voltammetry (b) and galvanostatic charge–discharge measurements (c). Reproduced from ref. 270. Copyright 2018 by Wiley‐VCH.

In cases where they are insoluble in the chosen electrolyte, some small molecules may be used without immobilization. Alizarin, which can be extracted from the plant *Rubia cordifolia* L and may also be used in supercapacitor applications,^268^, ^269^ is a prominent example of an anthraquinone‐like chemical that fulfils this requirement as it shows rather low solubility in water.^270^ By using an antisolvent approach, different morphologies of alizarin crystals may be obtained, with nanowires showing the best charge storage properties. Even at fast charge–discharge rates of 10 A g^−1^ with a sodium‐ion‐containing aqueous gel electrolyte, capacities of the half‐cells in the range of 135 mAh g^−1^ could be obtained (unfortunately, at which cycle this capacity was obtained was not reported). Partial dissolution in the electrolyte however resulted in poor cycling stability (57 % capacity retention as measured by cyclic voltammetry at a scan rate of 100 mV s^−1^ after 50 cycles). Nevertheless, full‐cell performance in a sodium‐ion battery setup with a polypyrrole‐based counter electrode was also demonstrated, showing rather promising performance (Figure 12).

Purpurin, which may be extracted from the plant *Rubia tinctorum* and which also has an anthraquinone‐like structure, has similarly been used as an active material in biogenic batteries.^271^ With slightly better cycling stability than alizarin, such electrodes may be appealing for future sustainable battery applications. Together with similar natural anthraquinones, such as rhein or sennidin B, emodin and alizarin have also been applied as anolytes in organic redox flow batteries,^206^, ^272^ in which immobilization of the active compound of low molecular weight is not important. In a fully organic redox flow battery with a catechol‐like catholyte, voltages in the range of 0.8 V are achievable. Studies concerning the cyclability and stability are ongoing.^206^

##### 2.2.2.4 Other biomass‐based quinones

**Figure 13:**
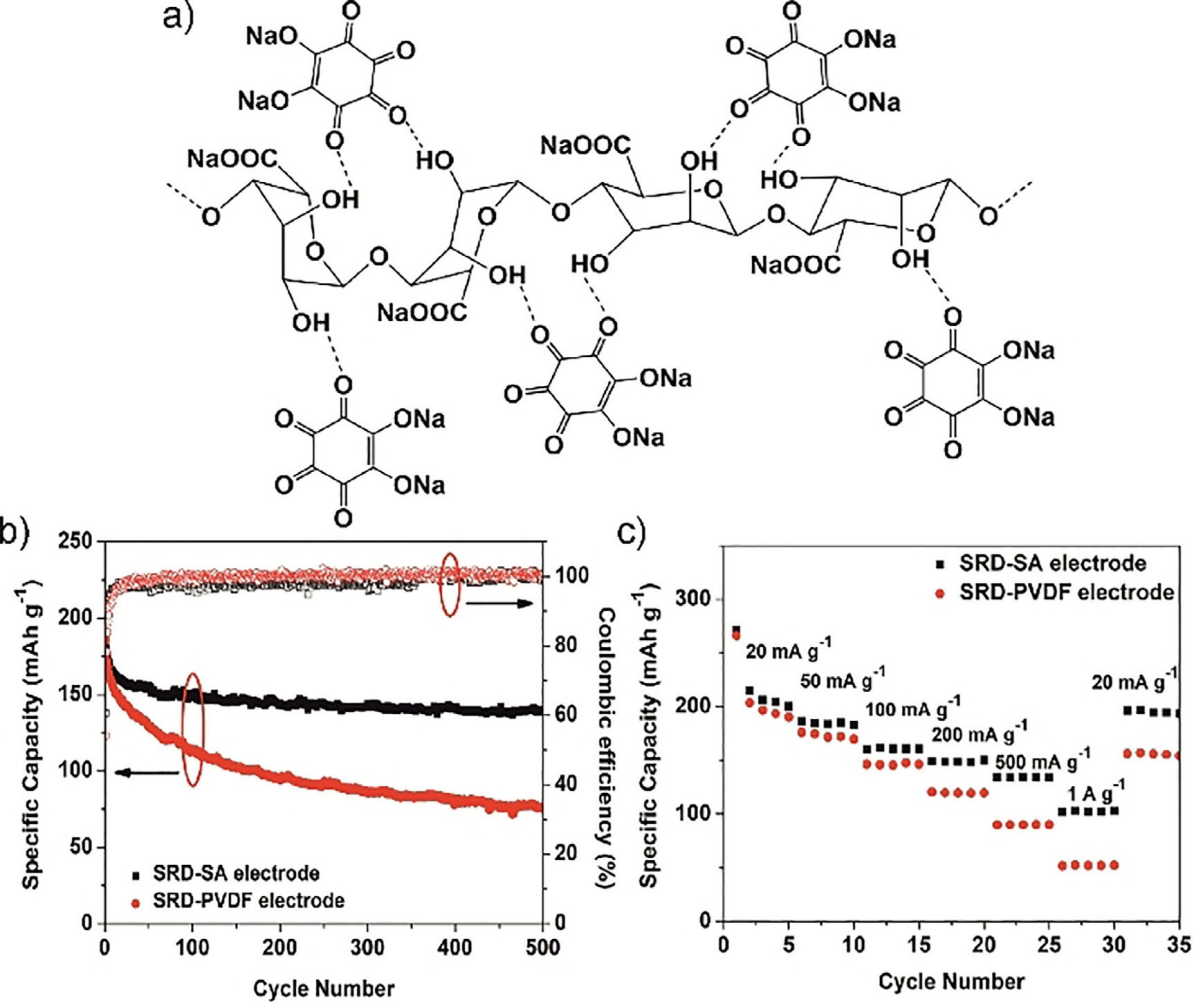
Rhodizonate‐based active material (SRD), which is used in combination with a sodium alginate (SA) binder. a) Schematic representation of the structure. b) Cycling performance in comparison to combinations of SRD with a PVDF binder. c) Rate performance of these electrodes. Reproduced with permission from ref. 277. Copyright 2017 by Elsevier.

Dilithium rhodizonate Li_2_C_6_O_6_, synthesized from *myo*‐inositol,^273^ was one of the first examples of using a bioderived molecule as redox‐active material for high energy storage.^1^ It has a redox potential in the range of 2.8 V vs. Li^+^/Li and can store up to four lithium ions. With a specific capacity of 580 mAh g^−1^ and an energy density of 1100 Wh kg^−1^ it is amongst the best performing sustainable cathode materials. Besides dilithium rhodizonate, also disodium rhodizonate and dipotassium rhodizonate have been described by several groups in the following in manifold compositions and surroundings.^127^, ^274^, ^275^ Often, however, capacity is decaying fast upon cycling, even at slow discharging rates, demonstrating the need for immobilization of small redox‐active biomolecules as discussed above. Interestingly, by using a Mg–Li dual‐salt electrolyte and magnesium anode, stability of rhodizonate cathodes was improved.^276^ In this setup, besides intercalation of lithium ions, reactions with magnesium ions and a substantial capacitive contribution to charge storage also increase the capacity and stability of the system. Another approach to significantly increase the stability of rhodizonate‐based electrodes is to use binders that strongly interact with rhodizonate both in the oxidized and reduced form.^277^ In this regard, Wang et al. realized very high stability by combining disodium rhodizonate with alginate binders and almost twice as high capacity after 500 cycles compared to a combination of the active biocompound with PVDF binders (Figure 13).

The reduced forms of rhodizonate—salts of tetrahydroxybenzoquinone—may be used similarly. Chen et al. reported the use of Li_4_C_6_O_6_ as a sustainable electrode material for lithium ion cathodes and anodes.^278^ It is similarly available from *myo*‐inositol or phytic acid and may be oxidized and reduced, making it usable in both electrodes of sustainable batteries. Capacity fading of almost 50 % within 50 charge–discharge cycles may be caused by the fact that the small molecular compounds were not bound but merely mixed with carbon and could dissolve in the electrolyte. Solid electrolytes may help to increase the stability. In this regard, Chi et al. investigated all‐solid‐state sodium batteries in which Na_4_C_6_O_6_ was the active component of both the cathode and anode.^279^ In this case, the capacity retention after 50 cycles was increased to 77 %, and probably mainly limited by volumetric changes upon charging and discharging, resulting in microcracks.

#### 2.2.3 Flavins and other pteridines

Flavins, another rather widely available group of redox‐active biomolecules feature similar redox potentials as quinones.^280^ They have not only been used in redox flow batteries^281^, ^282^ but also as solid electrodes in bioorganic batteries.^283^, ^284^ Because of the high polarity of the side groups, which enables easy dissolution in common electrolytes,^283^ steps towards increased stability are crucial in the latter. In this regard, combinations with carbon nanotubes may be advantageous, owing to strong π–π interactions between the two components.^284^ Furthermore, immobilization on polymer backbones may be employed.^285^

**Figure 14:**
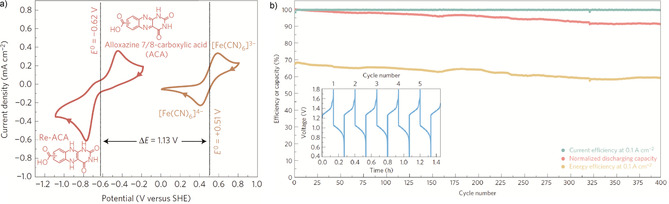
Cyclic voltammetry (a) and cycling performance (b) of a redox flow battery featuring alloxazine and ferrocyanide‐based electrolytes. Reproduced with permission from ref. 287. Copyright 2016 by SpringerNature.

Other pteridines, such as lumichrome, alloxazine, and lumazine, have subsequently also been investigated as redox‐active species for biomass‐based batteries, showing the high potential of flavins and related compounds for energy storage applications.^286^, ^287^ In light of the high solubility of pteridines, their application in redox flow batteries is especially appealing.^287^ Figure 14 shows such a setup featuring an alloxazine‐based electrolyte in combination with a ferrocyanide‐based counter electrolyte.

#### 2.2.4 Further redox‐active biomass‐derived materials in battery applications

In living organisms, a variety of biomolecules fulfils vital functions through reversible oxidation and reduction reactions. Besides quinone‐ and flavin/pteridine‐based molecules, other heteroaromatic compounds are present and have also been employed in battery applications. For example, nicotinamide adenine dinucleotide (NAD^+^) or uric acid may be used in redox‐flow batteries.^282^ Modified NAD^+^ was also employed as an active cathode material in sustainable batteries, exhibiting an average voltage of 2.3 V vs. Li^+^/Li.^288^

Finally, several electrode materials in organic batteries that have commonly been synthesized from petrochemical resources may also be obtained in elaborate processes from bioresources. The most prominent such molecule is aniline. It is the educt for making polyaniline, which can be used as a cathode material in organic batteries. Additionally, oligomeric^289^ or polymeric^290^ Schiff bases, which may serve as anode materials in organic batteries, can also be obtained from aniline derivates. Historically, aniline was obtained from indigo and hence from regrown biomass.^291^ Although, with the emergence of petrochemical industry, aniline production from indigo was soon completely replaced by petrochemical processes, nowadays aniline may also be produced on an industrial scale from biomass and hence may qualify as a truly sustainable chemical.^292^ Recent advances in aniline‐based electrode materials are summarized in reference 293.

### 2.3 Biomass‐based auxiliary materials in electrodes

Electrode formulation usually requires mixing of an active material, a conductive additive, and a binder in a mutual solvent or dispersion medium, formation of a slurry that can be spread onto a current collector, and post‐processing steps such as pressing or drying. Commonly, environmentally hazardous, reprotoxic, *N*‐methyl‐2‐pyrrolidone (NMP) is used as a solvent, and fluorinated binders are often incorporated. Moreover, conductive additives are often fabricated by energy‐consuming methods and may be derived from unsustainable starting materials. Even carbon‐based anodes and cathodes, as discussed above, are usually fabricated by mixing the active carbon material with a conductive carbon, such as carbon Super P, and a binder. Such additives often decrease the overall sustainability of the electrodes and may be exchanged by biomass‐based materials. As discussed above, studies have shown that biomass‐based carbons may replace conventional conductive carbons such as carbon Super P.^129^ Therefore, for truly sustainable electrodes, not only the active material but also the additives and solvents need to be adjusted.

PVDF is most often used as a binder for organic electrode materials and often constitutes as much as 10 % of the total electrode mass. It cannot be gained from bioresources and consequently lowers the degree of ecofriendliness of electrodes that incorporate it as a binder. Furthermore, it is hard to recycle and leads to toxic gases and harmful solid waste when burned.^294^ Consequently, in sustainable and biomass‐based battery materials, more benign binders should be used. Such biopolymeric binders have recently been reviewed by Nirmale et al.,^31^ Bresser et al.,^295^ and Li et al.,^296^ besides further recent reviews mentioning but not focusing on biogenic binders.^297^, ^298^, ^299^, ^300^, ^301^, ^302^ Being derived from biomass, they are often soluble in water, allowing for more benign production processes compared to electrodes that incorporate PVDF, because these are usually processed in solutions containing hazardous NMP. Switching to aqueous systems may result in shorter drying times at lower temperatures, increasing the overall sustainability of the battery materials.

Promising candidates shall be introduced in the following. Importantly, omission of any binders may be even more appealing than the use of biomass‐based binders as every binder decreases the relative amount of active material, so its addition possibly results in reduced capacity. Stability may instead be enhanced by crosslinking.^245^ Still, choice of the right binder may also result in enhanced interaction with the active material or electrolyte due to functionalities or changes in the electrode's polarity, respectively, and hence also increase the specific capacity.^246^, ^277^ Additionally, hydrophilicity of the binder material often facilitates ion mobility, permitting higher charge–discharge rates. Of course, the use of water‐soluble binders inheres limitations regarding the electrolyte solvent, as will be discussed below.

#### 2.3.1 Carboxymethyl cellulose and other cellulose‐based binders

**Figure 15:**
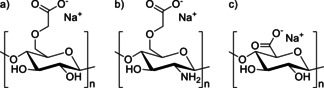
Structures of sodium carboxymethyl cellulose (a), sodium carboxymethyl chitosan (b), and sodium alginate (c). For simplification, different linkages between monosaccharides, different degrees of carboxymethylation (a,b), and different degrees of deacetylation (b) are not taken into account.

Cellulose‐based binders are the most prominent examples of sustainable binder materials,^303^ with carboxymethyl cellulose (CMC; Figure 15 a) being the most prominently studied derivative. This binder has been described for inorganic anode materials like graphite,^304^ silicon,^304^, ^305^, ^306^ hard carbons,^307^ tin/polypyrrole,^308^ MoS_2_,^309^ or TiO_2_,^310^ and is also applied commercially. When only a low amount of binder is used, particles of the active material are held together, whereas no blocking of ion pathways is observed.^304^ In particular, carboxymethyl cellulose with a high molecular weight and high amount of carboxymethyl groups exhibits promising binder properties.^306^

Carboxymethyl cellulose may also be used as a binder for inorganic cathode materials although this is not as well established as its application in anode materials. For example, in LiFePO_4_‐based cathodes CMC may be used instead of fluorinated binders.^311^, ^312^ Application in high‐voltage cathode materials, such as LiNi_0.4_Mn_1.6_O_4_,^313^ has also been reported, where CMC showed advantages in terms of reduced self‐discharge and higher capacity compared to PVDF as binder. Even in cathodes for lithium–sulfur batteries, CMC was used with better performance than PVDF or polyethylene oxide (PEO) binders.^314^

Importantly, even though some inorganic active electrode materials are vulnerable to the presence of water, short processing in aqueous slurries with CMC and conductive carbon may be acceptable, provided that the electrodes are thoroughly dried afterwards.^311^, ^315^ Thus, production of electrodes without the use of reprotoxic solvents like NMP is possible.

Unfortunately, however, CMC may also have detrimental properties. For example, noncovalent bonds with carbon‐coated LiFePO_4_ particles may decrease the electronic conductivity because of trapping of electrons within the carbon coating.^312^ In early cycles when using CMC as binder, acidic groups like ‐CH_2_COOH and ‐OH react with lithium to form ‐CH_2_COOLi and ‐OLi groups, respectively, decreasing the coulombic efficiency.^316^ The decomposition products contribute to the buildup of an SEI layer. Furthermore, a combination of CMC with petrochemical polymers is often used as a binder to optimize performance, albeit at the expense of the sustainability of this approach.^304^, ^314^ Thus, more research is necessary before such binders may replace less sustainable PVDF in the future.

In electrodes featuring an organic active material, only few studies describe the use of carboxymethyl cellulose as binder.^316^, ^317^ For example, anthraquinone‐based electrodes may be fabricated in a benign way in aqueous slurries using CMC binder.^316^ As discussed above, such a fabrication increases the sustainability of the battery and leads to lower production costs. The combination of CMC with biomass‐derived active materials would be the next important step towards truly sustainable electrodes.

Compared to other cellulose‐based binders, carboxymethyl cellulose features enhanced interactions with the active material (and thus enhanced binder properties), owing to the carboxyl group.^306^ Consequently, addition of more carboxyl groups in terms of grafted poly(sodium acrylate) side chains on CMC was employed to enhance binder properties in silicon anodes.^318^ Stronger interactions with the active material and current collector were observed.

Finally, cellulose and carboxymethyl cellulose were combined in paper‐like batteries, in which the anode, cathode, and separator all contained cellulose fibers.^319^, ^320^ The resulting batteries were rather stable, showed good mechanical properties, and still exhibited reasonable capacity.

#### 2.3.2 Chitosan

With a similar structure to cellulose and high natural abundance, chitosan‐based binders are a straightforward alternative to such binders. Comparably to carboxymethyl cellulose, consequently also carboxymethyl chitosan (Figure 15 b) was investigated as a possible binder for future electrodes.^321^, ^322^ In addition to the carboxylate functionality, amine functionalities may also support binding properties. Furthermore, functionalized chitosan‐based binders with different carboxylate^323^, ^324^ or amide^325^ side chains were introduced. Crosslinking of chitosan chains was shown to increase stability,^323^ and grafting natural rubber as side chains helped to increase flexibility.^326^

Usage of the same material in separators and binders can help inhibit sharp interfaces and thus enhance ion conductivity. Chitosan‐based materials were used for this purpose in supercapacitor applications, together with biomass‐derived carbon electrodes.^327^ Here, they were blended with poly‐ (ethylene glycol)‐*ran*‐poly(propylene glycol) to improve performance. With the binder and separator made from the same material, ion diffusion is more freely compared to chemically different materials.

#### 2.3.3 Alginates

Alginate‐based binders are structurally similar to cellulose‐based binders, but they include carboxylate groups in the native polymer structure and thus do not have to be modified with carboxylate‐containing side groups to develop their full potential (Figure 15 c). Consequently, they are facing increasing importance for battery applications featuring inorganic electrodes, both in anodes^328^, ^329^ and cathodes.^330^, ^331^ More importantly in terms of sustainability, however, are applications with organic or possibly even biomass‐derived active materials.^277^, ^332^, ^333^, ^334^

Recently, Luo et al. described the combination of a biomass‐based cathode material, sodium rhodizonate, with sodium alginate as a binder and thus the layout of a significantly more environmentally benign electrode.^277^ Functional groups in the active material and binder form strong interactions, resulting in significantly increased stability. Even during structural changes and rearrangements caused by electrochemical processes, reversible strong interactions lead to a self‐healing‐like mechanism, which prevents dissolution of the active material in the electrolyte.

Likewise, dilithium terephthalate as biomass‐based anode material was combined with sodium alginate binders.^334^ The electrochemical performance was significantly improved when compared to samples with PVDF binder, resulting from the facilitated ion transport through the electrode owing to the abundance of carboxyl and hydroxy functionalities.

**Figure 16:**
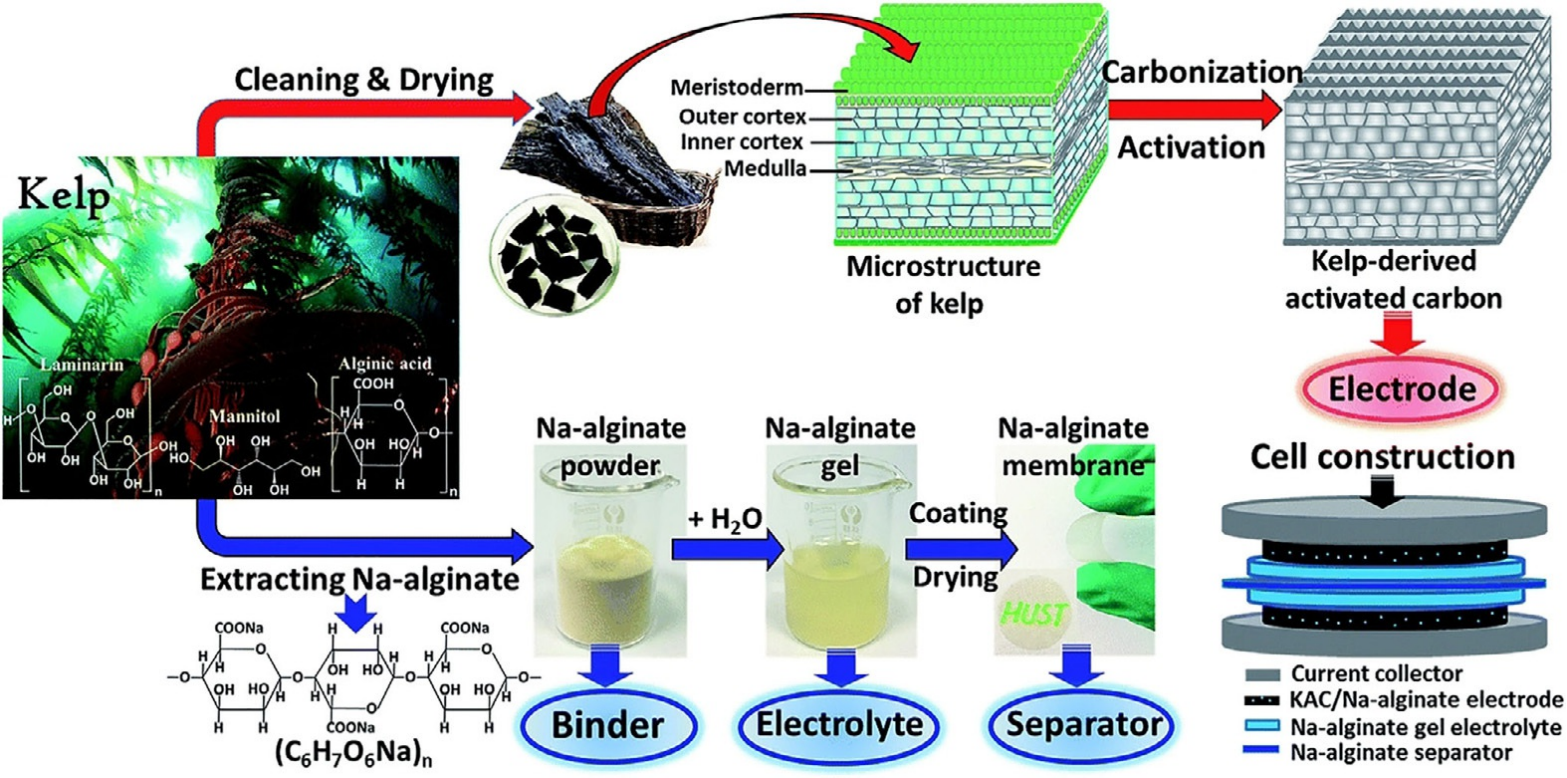
Process for the setup of all‐alginate‐based charge storage devices. Reproduced with permission from ref. 332. Copyright 2017 by the Royal Society of Chemistry.

As discussed above for carboxymethyl chitosan‐based binders, transport of ions may be facilitated if the separator and binder are made from the same material. In this regard, Zeng et al. used alginate not only as the binder in both electrodes but also in the electrolyte and separator of symmetrical supercapacitors, with carbon electrodes made from alginate, too (Figure 16).^332^ The reported device may be the best example of a truly biomass‐based energy storage device developed to date and highlights the importance of electrolytes and separators, as discussed in the second part of this Review.

#### 2.3.4 Further natural gums

Besides alginic acid, other polysaccharide gums have also been widely used as thickening agents, especially in the food industry. Several of these natural gums have recently also been employed as binder materials for more sustainable battery applications:

Similar to alginates, carrageenans are charged polysaccharides extracted from seaweeds. Instead of the carboxylate group, they carry ester sulfate groups on the repeating units of a linear polysaccharide backbone and may stabilize sulfur cathodes in lithium–sulfur batteries through possible reaction with polysulfides.^335^ Further naturally charged polysaccharides, such as karaya gum^336^ or gum arabic^337^, ^338^ have been used, as well as carboxymethylated polysaccharides, such as carboxymethyl gellan gum^339^ or carboxymethyl fenugreek gum,^340^ and processed polysaccharides, such as xanthan gum,^341^, ^342^, ^343^, ^344^ all of which feature carboxylic acid groups and thus provide binder properties mainly for anode active materials, as discussed above.

Finally, uncharged gums such, as guar gum,^345^ have found widespread application, both neat^346^, ^347^, ^348^ and in combination with other gums.^343^ The high density of oxygen‐containing functionalities makes such binders especially appealing for sulfur cathodes, as they may prevent polysulfide shuttling.^343^ Besides the absence of charged groups, guar gum features strong binding properties owing to its branched structure, which helps it to withstand severe volume changes during charging and discharging.

#### 2.3.5 Lignin

Lignin, which is among the most abundant biopolymers on Earth, also does not feature a linear but a branched structure. Although this structure may complicate good interaction and mixing with other constituents of the electrodes, especially at high molecular weight, it can also support binding properties. For example, upon grafting carboxylate functionalities to lignin, the branched structure helps to hold grains of active material together in the case of high volume changes upon charging and discharging.^349^

As discussed above, lignin has also been used as an active electrode material, owing to its redox activity after oxidation. Consequently, lignin binders may contribute capacity to an electrode and therefore not decrease the specific capacity of an electrode upon addition of the (lignin) binder. Instead, upon crosslinking, it may be used as an active material and a binder simultaneously.^245^ Its phenolic functionalities furthermore help to scavenge radicals, which may form in high‐voltage lithium‐ion batteries, improving their cycling behavior.^350^ In anodes of secondary lithium‐ion batteries, it may likewise be used, albeit without contributing additional capacity, owing to the mismatched electrochemical potentials.^351^ The extraction method and hence the exact structure and functionalities of lignin have only minor influences here. In contrast, the molecular weight matters, and low molecular weight fractions need to be removed for good binder behavior.^352^

#### 2.3.6 Proteins

Besides polysaccharides and polyphenols, proteins constitute one of the most important classes of natural biopolymers. In food industry, gelatin (denatured and hydrolyzed collagen) finds especially widespread application, owing to its binder properties. Consequently, it has also been employed as a binder for battery applications, where it shows comparable performance to other biopolymer binders.^353^ Its high dispersion and adhesion ability, together with electrochemical stability, make it a promising candidate for different cathode applications.^354^, ^355^, ^356^

Many other proteins have also been tested for binder applications in battery electrodes. For example, poly‐γ‐glutamate, which is present in fermented soybeans, may bind silicon and graphite particles in anode materials.^357^ Upon self‐assembly, soy protein binders or binders based on bovine serum albumin provide a stable environment with conductive pathways for ion transport.^358^, ^359^ Also in cathodes of lithium–sulfur batteries, soy protein binders may inhibit polysulfide shuttling.^360^ High‐voltage cathode materials may be stabilized by electrochemically stable silk sericin protein binders, which enable the formation of stable SEI layers.^361^ With the large variety of available proteins and their abundance of chemical functionalities and well‐defined structures, protein binders in general may be highly appealing for future applications. The same reasons however make the search for the best protein binder more difficult, and future research will show if a commercially applicable candidate will be found.

#### 2.3.7 Further auxiliary biomaterials in electrodes

Finally, it is important to mention that for some applications a combination of active material, conductive additive, and binder is insufficient. For example, printability of electrode materials in 3 D‐printed batteries may require high viscosity and shear‐thinning as desired properties, which may be enhanced by addition of nanocellulose to the printing ink.^362^

Even in the fabrication process of nonbiogenic electrodes, other vital biomolecules were also used. For example, ascorbic acid was used to reduce graphene oxide to be employed in more powerful lithium–sulfur batteries.^363^ Biomolecules may also serve as host for inorganic active species in electrodes.^364^ Furthermore, when incorporating auxiliary materials for heat dissipation into electrodes, a high surface area is highly valuable. In case of boron nitride, lignosulfonate may help in this regard during exfoliation.^365^ Similarly, lignin can be used to exfoliate molybdenum disulfide, incorporation of which in electrodes may facilitate electron and ion transport.^366^ Transport of the latter requires benign interactions with the electrolyte, a medium that will be described in more detail in the following section.

## 3 Electrolytes and Separators

During charging and discharging of an electrode, movement of cations or anions through the separator between electrolyte and electrodes compensates the change of charge. Being in contact with all other cell components, the electrolyte consequently is the most important when it comes to power density and safety considerations.^367^, ^368^ Especially when using organic active materials, solubility in the electrolyte may be an issue.^252^ Mainly depending on the cell voltage and composition of the electrodes, different electrolyte systems, composed of a salt in a solvent (or solid/gel matrix), may be used, and several reviews have described these different systems (without focusing on the environmental impact or production from biomass).^369^, ^370^, ^371^, ^372^, ^373^ However, besides the electrochemical stability and solubility of electrode materials, many other criteria, such as ion transport properties, liquidity range, viscosity, corrosivity, solid–electrolyte interphase (SEI) layer formation, and price, decrease the pool of practically applicable combinations to only a few. For example, lithium salts with fluorinated anions (mainly LiPF_6_) together with highly flammable carbonates are commonly used in electrolytes of lithium‐ion batteries.^370^, ^371^ The same applies to sodium‐ion batteries: most often, chlorinated or fluorinated sodium salts dissolved in carbonate‐based solvents are described.^374^ Although a large variety of additives can be added for example to reduce gas generation, protect against overcharging, or suppress flammability,^375^ the electrolyte remains a dangerous fluid.^8^

While most carbonates are produced from phosgene and thus ultimately from carbon monoxide (made from methane) and chlorine, ethylene carbonate and propylene carbonate are mainly made by the reaction of carbon dioxide with epoxides.^376^ The epoxides used for production are usually derived from oil‐based chemicals. Although carbonates may also be produced from biomass‐derived chemicals in elaborated multistep reactions, carbonate‐based electrolytes shall not be discussed here in more detail. Instead, the reader is referred to some excellent reviews.^369^, ^371^, ^374^, ^377^ In the following, more benign, biomass‐based, electrolytes shall be described.

### 3.1 Aqueous electrolytes

Firstly, however, aqueous electrolytes will be briefly discussed, as the most benign solvent for electrolytes is water, give that it is by far the most abundant liquid on Earth. In terms of environmental impact, aqueous acidic electrolytes with SO_4_^2−^‐ or PO_4_^3−^‐based electrolytes may be considered sustainable, as both sulfur and phosphorus are abundantly available on Earth.^21^ However, as the potential range of aqueous systems is limited, mainly low‐voltage batteries have been realized, with lead acid batteries being the most prominent example.^378^ Recently, aqueous zinc batteries with organic cathodes have also received increasing attention.^379^ As alkali metals react vigorously with water, the use of water in the electrolyte of high‐voltage batteries however is not intuitively recommended. Even so, as presented in several reviews, lithium‐based batteries may also operate in aqueous electrolytes under certain conditions:^380^, ^381^, ^382^, ^383^, ^384^, ^385^

One way to circumvent these issues is by separating alkali metal anodes from the aqueous electrolyte by a stable protection layer, for example in the form of a ceramic membrane^17^, ^381^ or graphene.^386^ On the cathode side, besides conventional cathode materials^385^ also oxygen may be used as reducible species without protection layer. Acidic or basic aqueous lithium‐containing electrolytes are being investigated in aqueous lithium–oxygen batteries, with the latter having the disadvantage of limited solubility of LiOH and precipitation issues, leading to blockage of pores in the case of lithium–oxygen batteries.^387^ In both cases, the necessary membrane to prevent contact between lithium metal and the aqueous electrolyte, together with an auxiliary organic electrolyte to enable lithium ion transport from the anode to the membrane and to protect the membrane from reduction decrease the overall ionic conductivity of the electrolyte and necessitate complicated setups and higher costs of the final cells.^381^ Consequently, conventional aqueous electrolytes find only limited application in lithium‐based batteries.

**Figure 17:**
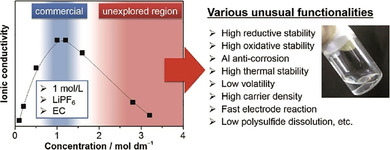
Properties of water‐in‐salt electrolytes. Reproduced with permission from ref. 388. Copyright 2015 by Yuki Yamada and Atsuo Yamada, published by the Electrochemical Society.

If however the concentration of lithium salt is increased to a range in which the molality of hydrated salt is higher than that of free water (i.e., when water is the minority component), such systems are denoted water‐in‐salt (WIS) systems, with their properties significantly deviating from conventional aqueous electrolytes (Figure 17).^388^ Wang, Xu, and co‐workers first showed that in this case the electrochemical stability range of water is drastically enhanced to approximately 3.0 V [in the case of water‐in‐Li bis(trifluoromethane)sulfonimide (TFSI) electrolytes].^389^ The increased stability is, to some extent, due to the decomposition of the TFSI anion on the lithium anode, creating an SEI layer and protecting the water from electrochemical decomposition.^390^, ^391^ Upon addition of another salt together with LiTFSI, the stability may be further tuned^392^ and, upon addition of SEI‐forming additives, high‐voltage electrodes may also be used.^393^, ^394^ Without artificially enhancing the electrochemical stability window, the voltage range is still sufficient for more sustainable (but lower voltage) electrode materials, enabling the production of rather “green” full lithium‐ion batteries.^395^, ^396^ Furthermore, the concept of water‐in‐salt electrolytes is not limited to lithium‐ion batteries but can be transferred easily for example to lithium–sulfur,^397^ sodium‐ion,^398^, ^399^, ^400^ or magnesium‐ion batteries.^401^

**Figure 18:**
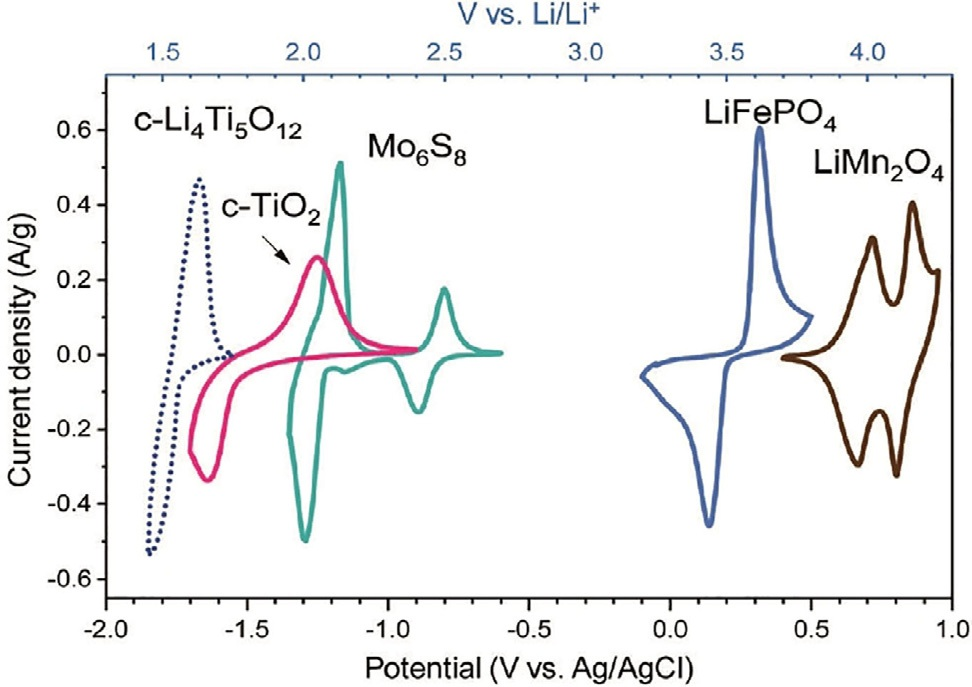
Cyclic voltammetry profiles of various active electrode materials collected in a mixed WIS electrolyte of 32 m KOAc and 8 m LiOAc. Reproduced with permission from ref. 404. Copyright 2018 by the Royal Society of Chemistry.

Unfortunately, high concentrations of fluorinated lithium salts not only drastically increase the price of the resulting batteries; the environmental impact is also severely compromised. Hence, the use of more sustainable lithium salts is necessary for the development of “green” water‐based electrolytes. Other combinations of lithium salts and water may be advantageous, especially those featuring anions that are based on abundant elements, such as sulfate, nitrate, or phosphate.^7^ The concepts of creating an artificial SEI layer on the electrodes or concentrated electrolytes, which both enable larger potential ranges for aqueous electrolytes, have also been successfully applied for lithium‐ion batteries employing such electrolytes.^402^, ^403^ In this regard, Bao and co‐workers presented WIS electrolytes featuring mixed acetates as salt species and thus a combination of biomass‐derived salts and water as electrolyte.^404^ Such electrolytes enable reversible redox reactions of common electrode active materials (Figure 18) and thus are a promising step towards future safe and sustainable electrolytes in commercial application.

### 3.2 Organic electrolytes

#### 3.2.1 Bioderived organic electrolytes (nonionic liquid)

Although aqueous electrolytes are being investigated as sustainable alternatives to common electrolytes based on organic solvents, the latter are still used in the vast majority of studies. Unfortunately, as they are commonly derived from petrochemical precursors, they usually do not qualify as sustainable electrolytes. Besides some ionic liquids (described below), there are however a few exceptions.

**Figure 19:**
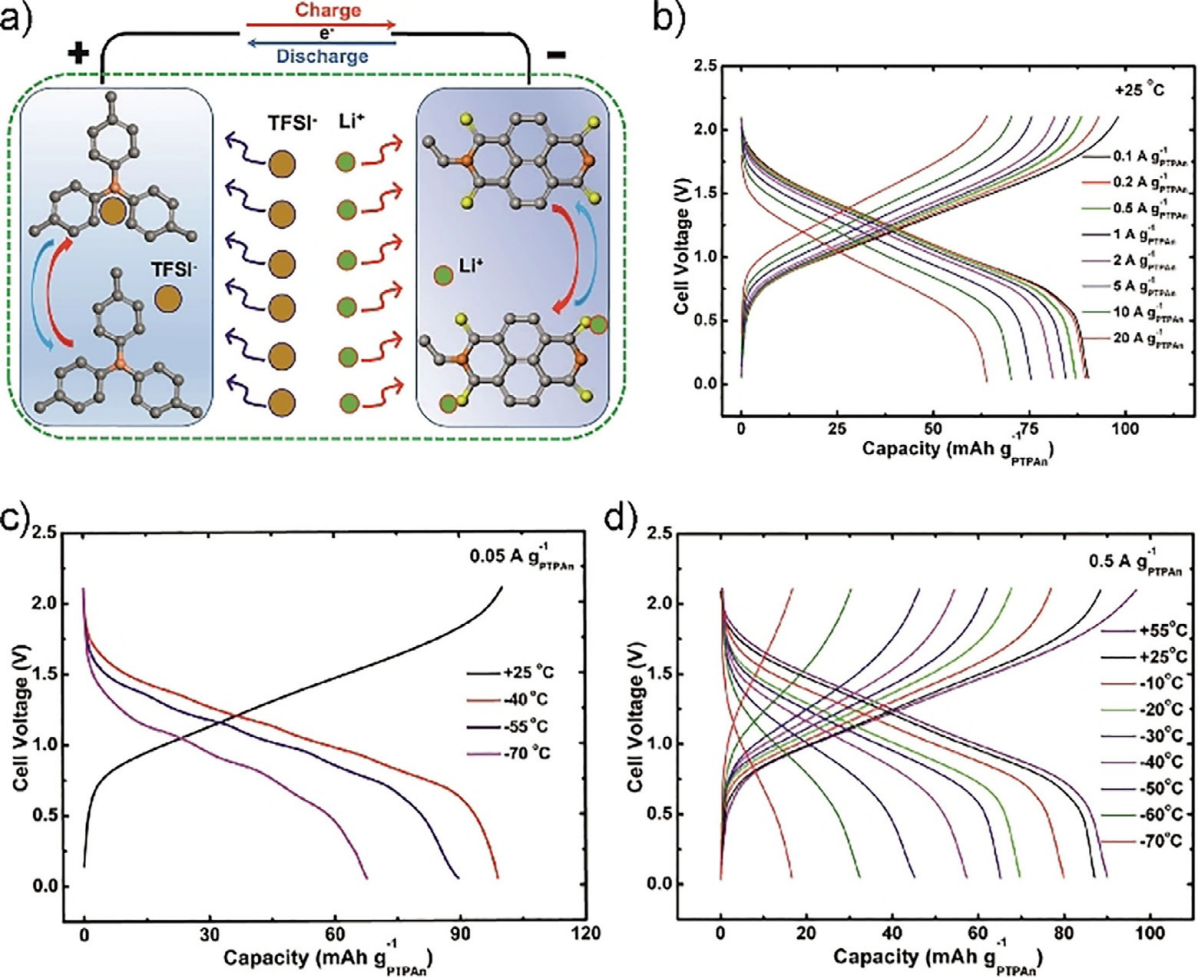
Setup (a) and electrochemical performance (b–d) of an electrolyte based on lithium TFSI dissolved in ethyl acetate at room temperature (b) and different temperatures (c, d). Charging before investigating the performance was performed at room temperature. Reproduced with permission from ref. 405. Copyright 2018 by Elsevier.

Recently, Dong et al. described ethyl acetate as the solvent component of electrolytes for lithium‐ion batteries, which were operable in a wide temperature range between −70 °C and +55 °C, in combination with organic electrode active materials (Figure 19).^405^ With ionic conductivities in the range of 0.2 mS cm^−1^ at temperatures as low as −70 °C and almost 1 mS cm^−1^ at −30 °C, an electrolyte based on LiTFSI dissolved in ethyl acetate could be used under extreme temperatures. At the low end of the investigated temperature range, some inorganic electrode materials could not be employed, so the authors also studied the applicability of lithium metal electrodes^406^ and the use of this electrolyte in an all‐organic setup with polymeric electrodes.^405^ Therefore, this approach is an important step towards more sustainable batteries.

#### 3.2.2 Sustainable ionic liquid‐based electrolytes

Ionic liquids (ILs) are often praised as green and sustainable solvents. Because of their low flammability, the tuneability of their properties, and their ionic nature, they often comply with several of the 12 principles of green chemistry described by Anastas and Warner.^4^ Depending on their structure and composition, they may be nontoxic, nonflammable, benign solvents, catalytically active, and make derivatization (like protecting groups during synthesis) unnecessary. However, when more clearly assessing their risk profiles in terms of the five risk indicators—release, spatiotemporal range, bioaccumulation, biological activity, and uncertainty—defining all ionic liquids as benign is not appropriate.^407^ The uncertainty is especially high, as many properties are simply not yet known.^408^ Although some ionic liquids may possess low risk, others have been found to be harmful or even rather toxic,^409^ especially many aprotic ionic liquids.^410^ Concerning sustainability, the synthesis of ionic liquids is usually not performed in a benign way, and their disposal or recycling often comes with unclear hazards resulting from the novelty and diversity of this class of fluids.^411^ Moreover, many of the properties that may define ionic liquids as green solvents (e.g., being a benign medium for organic synthesis or having catalytic properties) are of minor importance in battery electrolytes. Finally, ionic liquids in battery electrolytes are often halogenated compounds and based on anions such as PF_6_^−^, BF_4_^−^, TFSI^−^, or bis(fluorosulfonyl)imide (FSI^−^). Decomposition products, for example upon battery failure, include toxic, flammable, and ecotoxic substances such as fluoroethanol ether (FCH_2_CH_2_)_2_O, hydrofluoric acid (HF), and fluoromethane CH_3_F,^7^ and some ionic liquids are actually significantly more dangerous than conventional carbonate‐based solvents.^412^

**Scheme 2:**
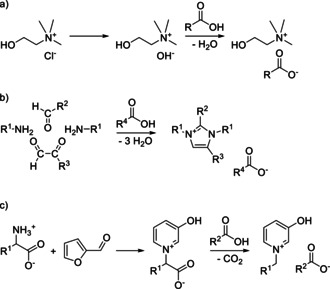
Synthesis of sustainable ionic liquids made from only renewable biomass. a) Cholinium, b) imidazolium, and c) pyridinium ionic liquids.

In contrast, there are also ionic liquids that may still be considered sustainable even when taking synthesis and decomposition products into account. Synthesis may especially be considered benign if both the cation and anion can be obtained from regrown biomass. In this regard, the most straightforward way for making biomass‐based ionic liquids is by combining choline, which, for example, is present in cell membranes, with a suitable anion or rather by exchanging the chloride or hydroxide in cholinium chloride or cholinium hydroxide with another bioderived anion by deprotonating a carboxylic acid^413^, ^414^ or amino acid^415^, ^416^, ^417^, ^418^ (Scheme 2 a). Although cholinium chloride itself does not qualify as an ionic liquid, owing to a high melting point resulting from the strong linkage between cation and anion,^418^ ionic liquids with glass transition temperatures down to −70 °C and decomposition temperatures up to 220 °C are available. Depending on the structure, completely bioderived cholinium ionic liquids with low toxicity are also available,^414^, ^417^, ^419^ making cholinium ionic liquids the role model for sustainable ionic liquids (or, rather, possibly sustainable, as commercially available choline is usually produced from unsustainable chemicals^420^).

**Figure 20:**
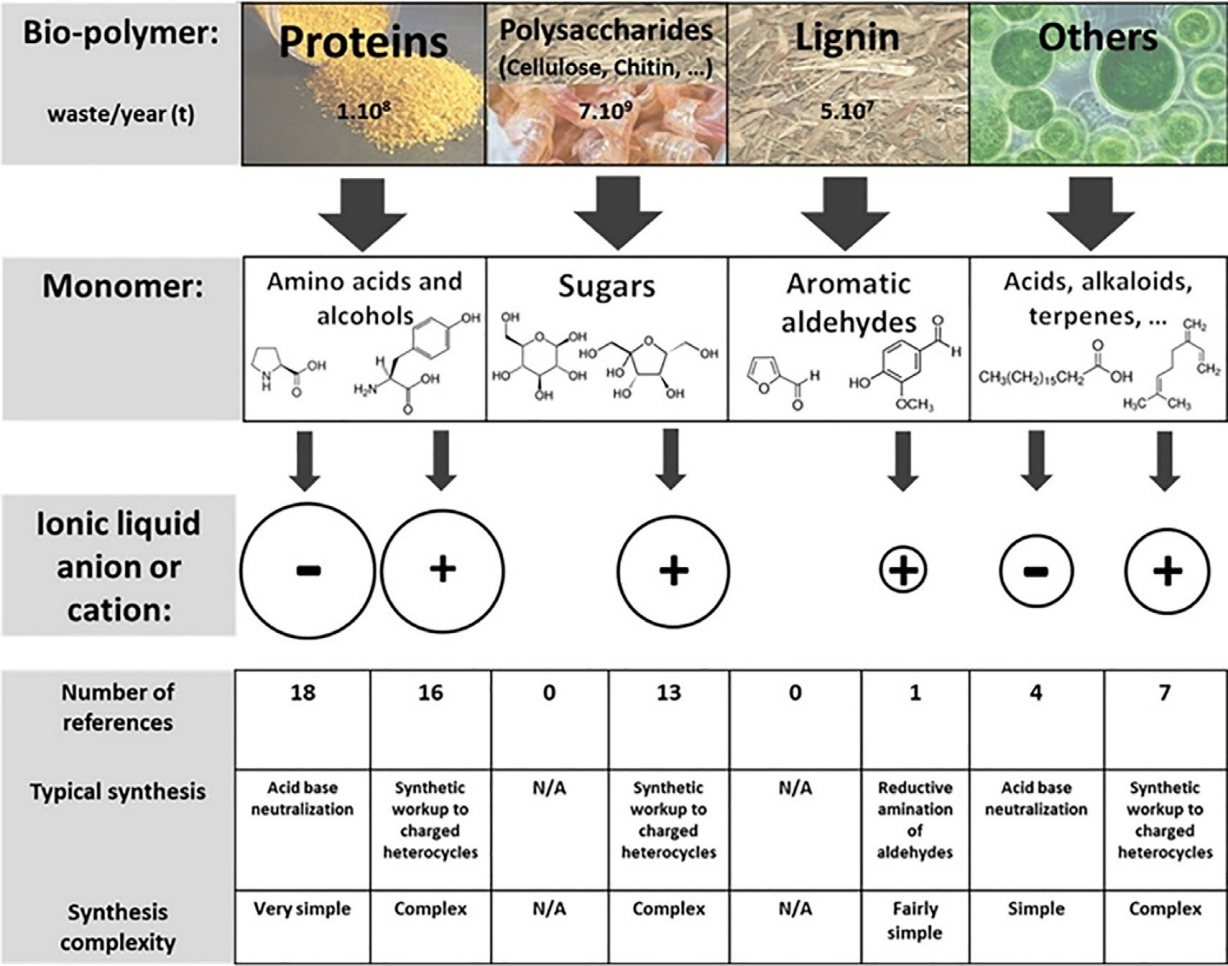
Synthesis pathways towards biomass‐based ionic liquids from different kinds of biomass. Reproduced with permission from ref. 421. Copyright 2016 by the American Chemical Society.

Besides using choline, a wide range of other ionic liquids from biological building blocks or including at least some biomass‐based components can be synthesized, as nicely summarized by Hulsbosch et al.^421^ In short, by using a complex synthetic workup, positively charged heterocycles are available for example from protein biowaste, polysaccharides, lipids, or even lignin‐based molecules, or they incorporate functionalities derived from such bioresources. Biomass‐based anions are more readily available, for example upon metathesis of the anion of an existing ionic liquid or other salt by carboxylic acids, fatty acids, or amino acids. Figure 20 summarizes the different routes leading to biomass‐based ionic liquids.

##### 3.2.2.1 Biomass‐based ionic liquids prepared by alkylation reactions

Synthesis of biomass‐based protic ILs is usually achieved by pretreating or processing the respective biomass to yield primary, secondary, or tertiary amines, protonating them by strong acids, and exchanging the anion by metathesis reactions to the desired counterion.^422^, ^423^, ^424^ Similarly, by using quaternization reactions instead of strong acid treatment with the other reaction steps being the same, biomass‐based aprotic ionic liquids are available.^425^, ^426^, ^427^, ^428^, ^429^

Amino acids are especially appealing as starting materials because not only anions, in the form of carboxylates, and cations, in the form of ammonium, are easily available (without first introducing amine functionalities), but also a variety of ring‐forming reactions may lead to oxazolium, thiazolium, or imidazolium type ionic liquids.^420^, ^430^ In this regard, chiral oxazolinium‐type ionic liquids can be obtained by alkylating oxazolines, which are available from the reaction of a chemically reduced amino acid with an aliphatic acid.^425^ When a dithioester is used instead of the aliphatic acid, thiazoliniums are available by a similar approach.^431^, ^432^ Imidazolium‐type ionic liquids are available by alkylating imidazoles, which may be formed from the reaction of an amino acid, ammonia, formaldehyde, and glyoxal in alkaline solution.^433^, ^434^

##### 3.2.2.2 Biomass‐based ionic liquids without the need for alkylation reactions

All these synthetic approaches for aprotic ionic liquids have in common that quaternization reactions, which are usually performed with rather unsustainable alkylation agents, are necessary. Although amino acids or saccharides have also been described as precursors for alkylation agents,^435^, ^436^, ^437^ their synthesis does not proceed via any more sustainable routes. Consequently, not all ionic liquids made by incorporating sustainable building blocks into their structure should be considered sustainable. Resource‐efficient and green reactions are necessary to justify this definition. Especially appealing in terms of sustainability are consequently only those ionic liquids that can be synthesized without any alkylation reaction. Besides using choline, as described above, imidazolium‐438, 439, 440, 441 and pyridinium‐type^442^ ionic liquids are accessible via such elegant reactions (Scheme 2 b, c).

Arduengo first described the synthesis of symmetrically substituted imidazolium ionic liquids without the use of alkylation reactions (Scheme 2 b).^438^ Unsymmetrically substituted imidazolium ionic liquids with substituents A and B are only available as a mixture of A,A‐, B,B‐, and A,B‐substituted imidazoliums.^438^, ^443^ The reaction can be still conducted under benign conditions, for example, in a continuous‐flow microreactor,^444^ and it can also be used to crosslink (biomass‐based) polymers with pendant primary amines^445^, ^446^ or to form polyelectrolytes.^446^, ^447^, ^448^ Conduction in aqueous environment at room temperature further adds to the benefit of this synthesis. Concerning the definition as a green synthesis, Esposito et al. showed that this reaction is also possible with stoichiometric amounts of all‐biomass‐based reagents.^439^ In this approach, two equivalents of an amino acid react in good‐to‐excellent yield with one equivalent of aldehyde and one equivalent of dicarbonyl, catalyzed by acetic acid. The products are solid zwitterions that can be either protonated by strong acids^439^ or decarboxylated in the presence of an acid in a benign hydrothermal reaction,^449^ leading to actual ionic liquids with the corresponding base forming the counterion.

**Figure 21:**
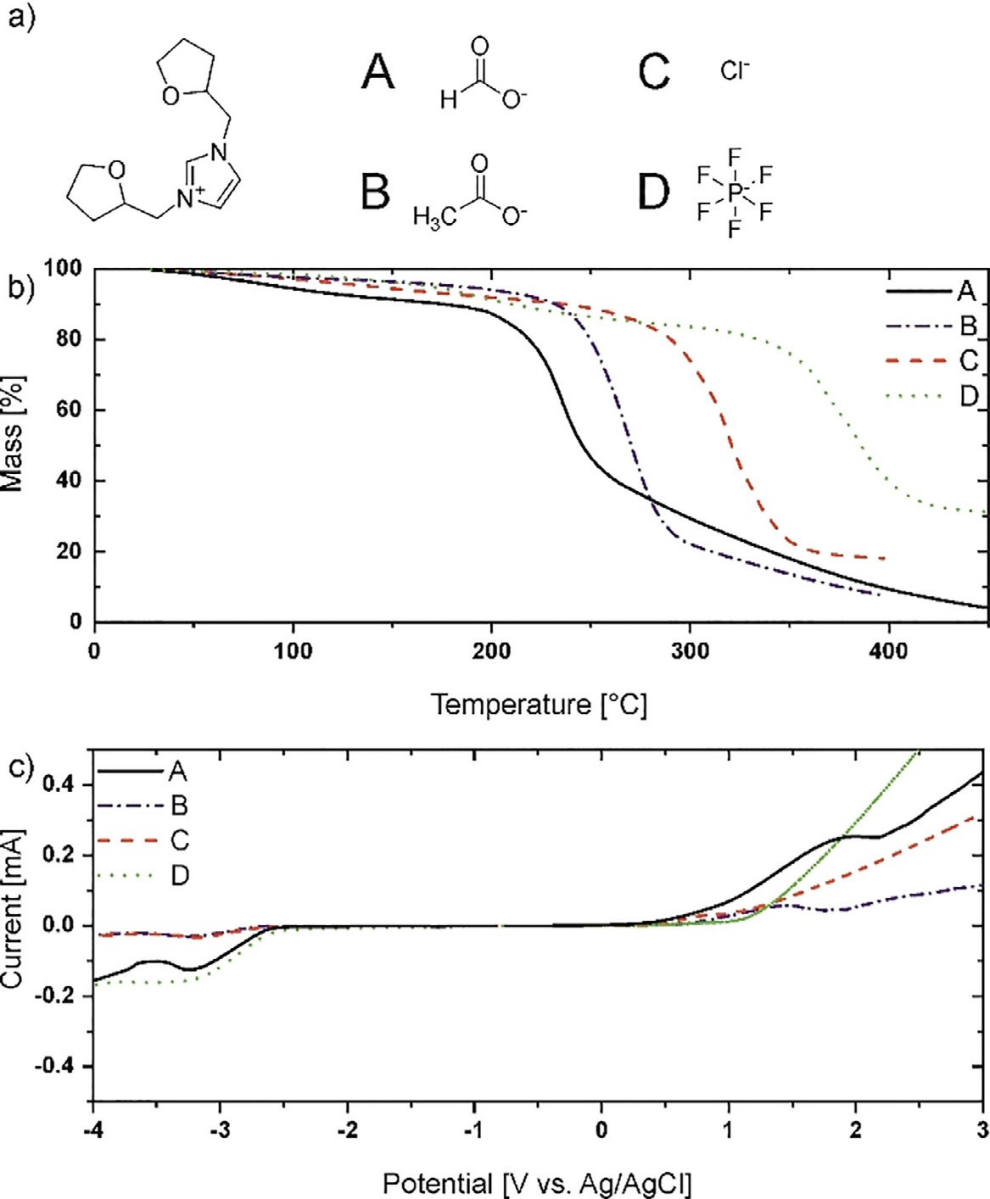
Sustainable imidazolium ionic liquids made from biomass‐derived chemicals. a) Structures. b) Thermal behavior. c) Electrochemical behavior. Adapted from ref. 441. Copyright 2017 by Wiley‐VCH.

Liedel et al. further elaborated on the sustainability and versatility of this approach.^441^ The authors showed that, besides amino acids, different saccharide‐derived amines, for example, can also be used. Ionic liquids with a vast variety of counterions could be synthesized in benign reactions by performing the ring formation in different diluted organic mono‐ or diacids. The choice of acid not only influenced the yield (excellent for weak acids) but also the liquidity and thermal stability, which could further be tuned by anion metathesis.^440^, ^441^ Figure 21 summarizes the obtainable ionic liquids together with their thermal and electrochemical behaviors. Interestingly, by using this approach not only is there no need for alkylation reactions, but also no hydrothermal decarboxylation or anion exchange is necessary, leading to truly benign synthesis conditions. Even more importantly, in the context of this Review, the authors also investigated the electrochemical stability and found it to be in the expected range for imidazolium ionic liquids (see Figure 21 and below).

Esposito et al. also synthesized pyridinium ionic liquids by a similar approach (Scheme 2 c).^442^ Fructose‐derive furfural reacted with an amino acid in a flow chemistry setup to yield pyridinium zwitterions. Different natural carboxylic acids introduced during subsequent hydrothermal decarboxylation resulted in pyridinium ionic liquids with a variety of different counterions, which in turn influenced the thermal behavior.

Unfortunately, truly sustainable ionic liquids, as described above, have not widely been described for battery electrolytes. The reason is that the oxidative and thermal stability of sustainable ionic liquids, which are free of halogens, are often lower than those of specially designed ionic liquids with fluorinated groups. Furthermore, even such high‐performance ILs have some disadvantages compared to carbonate‐based solvents (they are usually more viscous and have lower ionic conductivities, owing to sluggish diffusion of larger ion clusters).^374^ It is worth noting that the limited oxidative stability of sustainable ionic liquids is not limited to carboxylate anions, but also ILs with different other halogen‐free anions possess limited oxidative stability.^450^ Additionally, the interplay between all battery components of conventional batteries has been continuously optimized, making it difficult to exchange one species with a chemically very different one (e.g., a carbonate‐based solvent with an ionic liquid in general and a sustainable ionic liquid in particular).

One further property that impedes application of many ionic liquids in electrolytes is, ironically, often their high range of electrochemical stability. Modern lithium‐ion batteries require the formation of an SEI layer on the electrodes by partial electrochemical decomposition of the electrolyte system. A high reductive stability of an ionic liquid may prevent this formation, which in turn increases safety concerns rather than decreasing them.^371^ In this regard, biomass‐based ionic liquids may be more appealing for actual applications than other ionic liquids as their range of electrochemical stability is often smaller than in other ionic liquids but still comparable to some organic solvents. For example, 1,3‐bis[(tetrahydrofuran‐2‐yl)methyl]imidazolium acetate, which is a completely bioderived symmetrical imidazolium ionic liquid with a tetrahydrofurfuryl group as each *N*‐substituent and an acetate counterion, has an electrochemical stability range of approximately 3.6 V and is not stable vs. lithium metal (as most imidazolium ionic liquids in which no protecting group is introduced at the carbon between both nitrogen atoms).^441^ As such, it may be suitable in electrolytes of secondary lithium batteries but has not been investigated yet, as is also the case for any imidazolium ionic liquid synthesized in the sustainable manner described above.

It has to be noted that in sustainable imidazolium ionic liquids, the introduction of protecting groups at the carbon atom between the two nitrogen atoms (R^2^ in Scheme 2 b), which would lead to increased reductive stability, is difficult by the sustainable synthesis route described above (R^2^ is usually a proton).^440^ If a higher reductive stability is necessary, for example in batteries in which an SEI layer must not be formed (e.g., in magnesium batteries^451^), such ILs may consequently not be used as such. To circumvent this issue, a protecting group may be introduced after synthesis, for example upon formation of an N‐heterocyclic carbene (NHC) borane in an easy way.^452^ The resulting zwitterions indeed show superior reductive stability^453^ and have recently been described as the solvent component in the electrolyte of magnesium batteries.^454^ Applying this approach of forming an NHC borane to truly sustainable imidazolium ionic liquids may consequently also lead to the use of sustainable imidazolium‐type ionic liquids in electrolytes for magnesium‐ion batteries.

##### 3.2.2.3 Outlook for biomass‐based ionic liquid electrolytes

Especially sustainable cholinium ionic liquids have been applied in the electrolytes of modern batteries. For example, Jia et al. demonstrated the application of cholinium nitrate embedded in the biopolymer chitosan as an electrolyte in biocompatible magnesium–air batteries.^455^ Within this ionogel electrolyte, the ionic conductivity is higher than in the ionic liquid itself. Supplying a volumetric power density of 3.9 W L^−1^, the battery employing this sustainable electrolyte would be sufficient for powering some cardiac pacemakers or biomonitoring systems.

**Figure 22:**
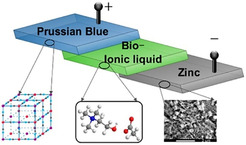
Setup of a sustainable Zn‐ion battery featuring a Zn anode, a Prussian blue cathode, and an ionic liquid electrolyte based on cholinium acetate. Reproduced with permission from ref. 456. Copyright 2016 by the American Chemical Society.

Although non‐bioderived cathode and anode materials were still used, a setup for a full battery composed of only sustainable components was presented by Liu et al., who described a rechargeable zinc‐ion battery (Figure 22).^456^ Together with a Prussian blue analogue as the cathode material and nontoxic zinc as the anode, zinc acetate dissolved in a mixture of cholinium acetate and water served as the electrolyte for this environmentally friendly battery. Zinc could be reversibly dissolved/deposited without dendrite growth and the full battery delivered a reversible capacity in the range of 120 mAh g^−1^ at a discharge rate of 10 mA g^−1^. Although the water‐based electrolyte and the use of zinc as anode limited the voltage of this battery to approximately 1.1 V, the presented battery is especially appealing in terms of sustainability because all components are cheap and abundantly available from nature or, in the case of the ionic liquid, from natural precursors.

As indicated above, some protic ionic liquids may be derived in a completely sustainable way from renewable resources, as no alkylation reactions are necessary. In general, protic ILs, although they contain protons, may be used in lithium‐ion^457^ or sodium‐ion^458^ battery electrolytes with sufficiently low water content and hence immobility of the protons. However, battery electrolytes based on protic ionic liquids in which both ions are available from bioresources have not been reported to date.

Finally, biomass‐derived ionic liquid crystals may be used as solid electrolytes in sustainable battery applications. Using a waste product of the cashew industry as reagent, Devaki and co‐workers introduced an imidazolium‐based ionic liquid crystal electrolyte for supercapacitor applications with excellent cycling stability^459^ or for lithium‐ion batteries.^460^ These or other biomass‐based liquid crystals may be appealing for future applications as solid electrolytes/separators. Solid/gel‐like electrolytes and separators made from biomass in general will be introduced next.

### 3.3 Bioderived solid and gel‐like electrolytes and separators

Separators in conventional lithium‐ion batteries are usually based on porous petrochemical materials, such as polypropylene and polyethylene. With high mechanical stability, despite low thickness, they reliably isolate the electrodes from each other and enable charge compensation due to the flow of ions through the membrane, usually with low selectivity.^461^ For high power applications, however, high transference numbers of lithium ions close to unity are desirable.^462^ Upon attaching functional groups like anions or Lewis acid groups, which trap anions from the metal salt in the electrolyte, such high transference numbers might be accessible.^463^, ^464^

Polymer membranes may simultaneously serve as separator and solid/gel‐like electrolyte. Besides conventional petrochemical polymers and designer polymers,^461^, ^463^, ^465^, ^466^, ^467^, ^468^ biopolymers have also recently been discussed as functional units in separators or as part of solid polymer electrolytes.^49^, ^203^, ^469^, ^470^, ^471^, ^472^ Besides mechanically separating the electrodes, biomass‐based solid electrolytes may fulfil further important tasks, such as prevention of polysulfide shuttling in sulfur‐based batteries.^473^, ^474^ Importantly, as discussed above, conduction of ions through interfaces will be facilitated if the chemical environment in the electrode material is similar to the environment in the separator or electrolyte. Consequently, it might be advantageous to make binders and separators from the same biogenic polymer material and thus prevent the formation of hard interfaces.^319^, ^320^, ^327^, ^332^

Gel‐like electrolytes may be even more appealing as they tend to exhibit better ionic conductivities than solid electrolytes, which is one of the biggest drawbacks of the latter. The mobile phase usually consists of a conventional electrolyte and thus has the same advantages concerning conductivity and drawbacks regarding stability, hazards, and sustainability as other electrolytes.^463^ Consequently, not only the use of biopolymers as matrix component of gel electrolytes is important for increased sustainability but also exchange of the mobile phase to a more benign liquid electrolyte. Sustainable ionogels might be beneficial in this regard.

Such ionogels can be formed by swelling a solid material (e.g., a polymer) in an ionic liquid. Being comparable to hydrogels only in that the mobile phase is an ionic liquid instead of water, ionogels are versatile materials with good transport properties and structural integrity. Both are important for gel electrolytes in batteries. In ionogel electrolytes for lithium‐ion batteries in which the counter anion of the lithium salt is the same as the anion in the ionic liquid, high lithium transference numbers may be reached. Ionic conductivity in such materials is usually comparably high, similar to polymer‐free liquid systems.^463^ However, there is still often a tradeoff between ionic conductivity and structural stability, which demands new materials and thorough physicochemical characterization.

**Figure 23:**
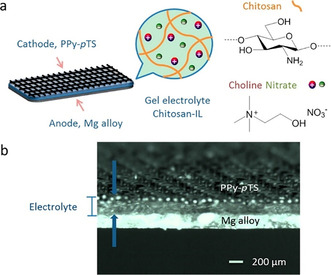
Schematic (a) and microscopic (b) images of a magnesium–air battery featuring a cholinium nitrate ionogel electrolyte. Reproduced with permission from ref. 455. Copyright 2014 by the American Chemical Society.

The combination of both approaches, that is, biopolymer gel electrolytes that use ionic liquids as the mobile phase, has been described for solar cell applications in the past.^475^, ^476^ Transfer of this approach to battery applications and use of truly sustainable ionic liquids, as discussed above, may be the next steps regarding sustainable gel electrolytes for sustainable energy storage applications. A first step in this direction was reported for a chitosan‐based matrix and cholinium‐type ionic liquid as the mobile phase.^455^ This electrolyte was used in combination with a magnesium alloy anode and polypyrrole–*para*(toluene sulfonic acid) cathode for magnesium–air batteries (Figure 23).

#### 3.3.1 Cellulose‐based materials

Materials based on cellulose may be especially promising, as cellulose‐based separators are already being commercially applied in alkaline batteries. Consequently, such separators have been investigated in combination with all kinds of electrodes, including not only inorganic but also organic electrode materials.^477^ However, limitations from high water content, stability problems, and safety issues have prevented commercial application in lithium‐ion batteries to date.^471^ Several researchers have tried to circumvent such issues, for example, by thorough drying.^478^ It is worth noting that the high water content may also be beneficial for fabrication from aqueous slurries, for example, in simple paper‐making processes.^319^, ^320^ Even more importantly, for supercapacitor applications that employ aqueous electrolytes, significant water content of the separator is irrelevant—in contrast, hydrophilicity here is advantageous for optimal ion transport.^479^, ^480^, ^481^, ^482^

**Figure 24:**
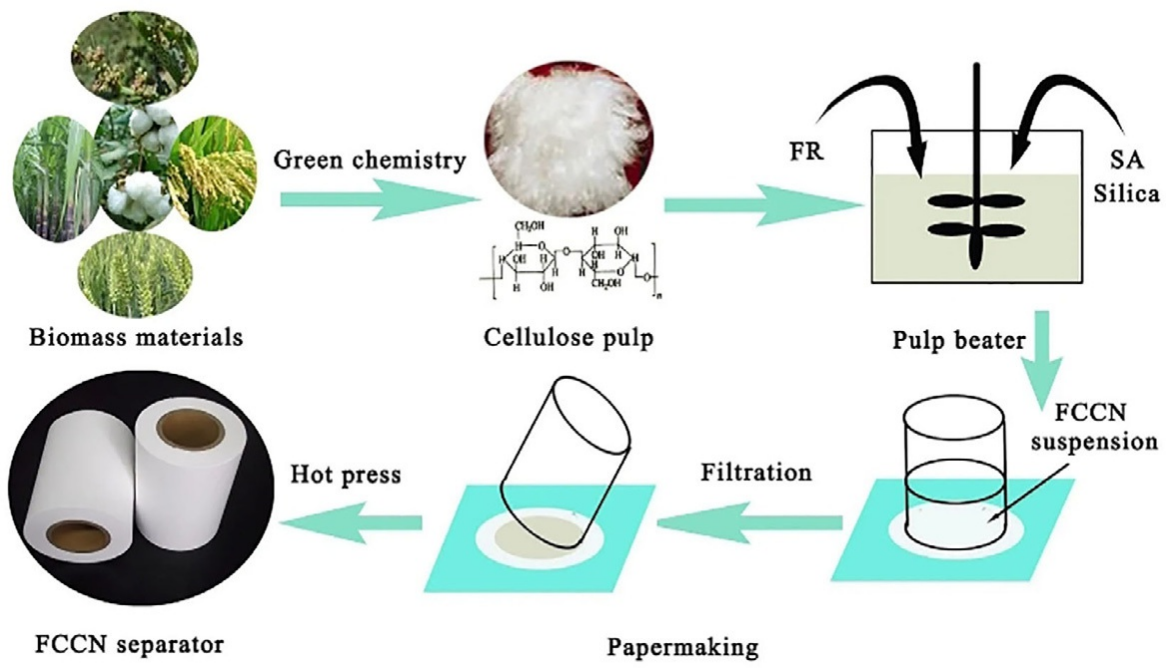
Process of making cellulose‐based separators by mixing cellulose pulp with sodium alginate (SA) and a flame retardant (FR), filtering the suspension, pressing, and drying it. Reproduced with permission from ref. 485. Copyright 2014 by SpringerNature.

When cellulose is used as a support for another separator material, the properties of that other material might dominate or, at the very least, influence the performance, mitigating the adverse properties of cellulose but simultaneously decreasing the overall sustainability in cases where the other material is not biomass‐derived.^477^, ^481^, ^483^ Composite or hybrid separators based on combinations of cellulose‐derived materials with other materials have been described, in which additives enhance the material's properties.^482^, ^484^, ^485^, ^486^, ^487^ Of these materials, combinations with other biopolymers, such as alginate,^485^ gelatin,^487^ and lignin,^481^ are especially appealing (Figure 24).

For high transference numbers, increased stability, and to prevent disintegration to nanofibers, negatively charged groups may be introduced on the surface of cellulose fibers,^486^, ^488^ for example, by conversion to carboxymethyl cellulose (CMC).^480^, ^489^ Consequently, besides the addition of additives, chemical modification is a widely employed tool to increase the performance of cellulose‐based separators/solid or gel‐like electrolytes. Interestingly, the protonated form of CMC showed highest cycling stability in lithium‐ion batteries.^489^ Besides CMC, methyl cellulose^490^ and cellulose acetate^482^ have also been investigated for use in cellulose‐derived separator/solid or gel‐like electrolyte setups.

#### 3.3.2 Other biopolymers in solid/gel‐like electrolytes and separators

Cellulose‐based solid/gel‐like electrolytes and separators are particularly appealing in terms of the abundance of the raw material, but their applicability without chemical modification or formation of composites is limited. Other biopolymers have consequently also been investigated in separators/solid or gel‐like electrolytes, sometimes also in combination with cellulose.^481^, ^485^, ^487^

Similarly to binder materials (see above), alginates are another important class of biopolymers that are used in electrolytes and separators.^332^, ^485^, ^491^, ^492^ In energy storage device that are completely derived from biomass, they may serve not only as a binder and precursor for carbon electrodes, but also as the gel electrolyte and separator.^332^ When combined with benign ion species and electrodes, batteries incorporating an alginate‐based separator may even be fully biodegradable and biocompatible, enabling the realization of in vivo power supplies.^491^

Chitosan may likewise be used as a component of the binder and separator to mitigate sharp interfaces and maximize ionic conductivity.^327^ For improved performance, it can be blended with another polymer. In general, similarly to cellulose‐based separators/electrolytes, blending or compositing is a widely employed approach to optimize performance. Combinations with inorganic filler materials or thermally stable polymers are especially appealing, as such combinations may be inherently stable and do not shrink upon heating, improving the safety of batteries by prevention of short‐circuit faults.^483^, ^485^, ^492^, ^493^ In general, solid or gel‐like electrolytes/separators are complex materials in which the exact composition often determines the applicability. Biomass‐based polymers may be an important piece in that big jigsaw puzzle.

## 4 Summary and Outlook

Currently, most organic battery materials are produced from petrochemicals. Furthermore, coal may also be used as a precursor material, with abundant supply.^378^ However, new biorefinery approaches have enabled the production of all kinds of raw chemicals from bioresources.^44^, ^494^ With more efficient and economic routes, all organic battery materials may potentially be available from biomass.

Nowadays, however, not all chemicals are readily available from bioresources. Besides common biopolymers, such as cellulose or lignin, which may find application in binders, separators, and solid or gel‐like electrolytes, several small molecules are being utilized industrially on such a large scale that production from biogenic precursors is feasible. Terephthalates are a prominent example of this kind of chemicals, and they have been investigated by many research groups as active materials in future anodes for lithium‐, sodium‐, or potassium‐ion batteries. Furthermore, several quinones, under investigation mainly as cathode materials, have been obtained from bioresources. Even ionic liquid electrolytes have been synthesized by using purely biomass‐derived chemicals.

Finally, carbonaceous materials are an important constituent of all kinds of batteries, not only as host materials for metal ions in anodes but also, for example, as conductive additives. Using all kinds of biowaste or biogenic chemicals, carbons have been synthesized with the possibility to tune properties such as porosity or conductivity. Consequently, basically all biomass on Earth may possibly find use in battery applications in the future, either in the form of biomass‐based specialty materials or as precursors for fine chemicals or carbons. Although it goes without saying that inorganic materials are often preferable when it comes to high‐voltage or high‐energy‐density applications, drawbacks in terms of their sustainability may be completely circumvented by switching to fully bio‐derived energy storage devices in the future.

## Conflict of interest

*The author declares no conflict of interest*.

## Biographical Information

Clemens Liedel is a research group leader at Max Planck Institute of Colloids and Interfaces, Germany, investigating sustainable energy storage materials. Before, he graduated from RWTH Aachen University, Germany, and became fascinated about polymeric battery materials during a stay as a Postdoc at Cornell University, USA. His main scientific interests are in the fields of biogenic polymer cathodes and biomass‐based ionic liquid electrolytes.